# Pooled prevalence, spatial variation and associated factors of risky sexual behavior among sexually active men and women aged 15–49 years in sub-Saharan Africa, 2015–2024: evidence from Demographic and Health Survey

**DOI:** 10.1186/s12889-026-28529-0

**Published:** 2026-07-15

**Authors:** Hana Melaku Gebeyehu, Rehana Essa Getu, Tigist Kifle Tsegaw

**Affiliations:** Department of Epidemiology and Biostatistics, Institute of Public Health, College of Medicine and Health Sciences, University of Gondar, Gondar, Ethiopia

**Keywords:** Risky sexual behavior, Sexually active, Men, Women, Sub-Saharan Africa

## Abstract

**Background:**

Risky sexual behavior remains a significant public health challenge in Sub-Saharan Africa, contributing to the high prevalence of HIV/AIDS, sexually transmitted infections. Despite extensive research on risky sexual behavior among specific populations such as adolescents and young women, there is limited evidence on the spatial variations of risky sexual behavior and its determinants among men and women aged 15–49 years across Sub-Saharan Africa.

**Objectives:**

This study aimed to assess the pooled prevalence, spatial variation, and associated factors of risky sexual behavior among sexually active men and women aged 15–49 years in sub- Saharan Africa using recent Demographic and Health Surveys data from 2015 to 2024.

**Methods:**

A community-based cross-sectional study was conducted using Demographic and Health Surveys data from 27 SSA countries. A weighted sample of 428,909 sexually active adults was analyzed. Spatial analysis techniques, including Global Moran’s I, Getis-Ord Gi*, and spatial interpolation, were used to detect clustering patterns. Spatial regression models such as Ordinary Least Squares and geographically weighted regression were employed to identify associated factors and assess spatial non-stationarity.

**Results:**

The pooled prevalence of risky sexual behavior in SSA was 17% (95% CI: 13%–20%), with significant variation across countries ranging from 3% in Senegal to 39% in Liberia. Spatial analyses revealed statistically significant clustering of risky sexual behavior (Global Moran’s I = 0.42, *p* < 0.001), in parts of Southern and Western Africa. Key predictors of risky sexual behavior included being male, not married, unemployed, media exposure, and having been tested for HIV. The influence of these predictors varied significantly across geographic regions, highlighting substantial spatial heterogeneity.

**Conclusion:**

Risky sexual behavior remains a major public health concern in SSA, with notable spatial variation and locally varying determinants. Targeted, geographically tailored interventions that address local socio-cultural and structural factors are important to effectively reduce risky sexual behavior and its health consequences in the region.

**Supplementary Information:**

The online version contains supplementary material available at https://doi.org/10.1186/s12889-026-28529-0.

## Introduction

Risky sexual behavior (RSB) is an act that raises the vulnerability of an individual to sexual and reproductive health problems. This contains having sex at an early age, involving with multiple sexual partners (MSP), and unprotected sexual behaviors [1]. Adults constitute the biggest sexually active population globally, with approximately 3.2 billion individuals at risk of sexually transmitted infections (STIs) due to risky sexual behaviors (RSB) [2]. RSB raises the risk of contracting a sexually transmitted infection (STI).

RSB is an important public health concern, contributing to the high burden of HIV/AIDS. Globally, STIs and HIV remain major public health concerns. Every day, more than 1 million STIs are being contracted worldwide, with sexually active adults bearing the highest burden [3]. In 2022, approximately 39 million people were living with HIV, with 1.3 million new infections and 630,000 AIDS-related deaths [4]. Sub-Saharan Africa (SSA) is the most affected region, accounting for 60% of new HIV infections in 2021 [3].

Unsafe sex is responsible for a massive global morbidity and disability; According to the Global Burden of Disease, unsafe sex contributed 570.8 disability-adjusted life years (DALYs) per 100,000 people globally in 2019 [5]. more than any other related cause. This burden exhibits gender disparities. Women and girls bear a disproportionate share of the consequences, accounting for 53% of all people living with HIV globally [6], a burden heavily intensified in sub Saharan Africa where they account63% of the new infections [6].

Overall, the World Health Organization notes that HIV, viral hepatitis, and STIs together cause 2.5 million deaths each year [7], driven largely by risky sexual behavior.

Geographically, sub-Saharan Africa shoulders the heaviest HIV/STI burden: Eastern and Southern Africa together account for 20.8 million of the approximate 39 million global HIV cases [8]. and unsafe-sex-related mortality is highest in southern Africa [5]. In contrast, regions such as Australasia have much lower unsafe-sex burden [5].

RSB contributes significantly to adverse sexual and reproductive health outcomes. Non-fatal STIs can lead to complications such as ectopic pregnancies, infertility, and cervical cancer [9]. Additionally, 25 million unsafe abortions occur annually, many resulting from unprotected sex [10]. Gender disparities further exacerbate risks, as women often lack decision-making power in sexual relationships, increasing their vulnerability to STIs and unintended pregnancies [11].

A study conducted among young adults in Tanzania, found that 62% of participants reported engaging in at least one form of RSB. Notably, 28% had multiple sexual partners, and 34% reported inconsistent condom use [12]. Another study in Nigeria 17% of men aged 15–49 reported having multiple sexual partners in the past year. Among these, 65% reported inadequate condom use during their last sexual intercourse [13]. And in Ethiopia 27.5% of youth practiced risky sexual behaviors [14].

Several socioeconomic and demographic factors have been found to be factors for these risky behaviors. Studies have found that lower educational attainment, younger age, and lack of stable partnerships are significant predictors of engaging in high-risk sexual activities among Adults in SSA [15]. Moreover, variations, such as place of residence, household wealth, and education level, have been associated with risky sexual behaviors in the region [16].

To Address this growing concern, multiple interventions Strategies such as sexual health education, improved access to contraceptive methods, promotion of HIV testing and counseling, and harm reduction approaches for substance use have shown effectiveness in reducing risky sexual practices [17]. Additionally, The Sustainable Development Goals (SDGs), particularly Goal 3, emphasizes the importance of universal access to sexual and reproductive health services in reducing STI transmission and improving overall sexual health outcomes by 2030. However, the progress remains slow due to this high burden of risky sexual behavior in the region [18].

There is a lack of comprehensive studies examining the spatial distribution of risky sexual behaviors among adult men and women in SSA. Therefore, understanding the geographical patterns and associated factors is essential for developing targeted interventions and policies aimed at reducing the spread of sexually transmitted infections and promoting sexual health in the region. Moreover, by conducting a spatial analysis, this study aimed to identify regional variations in risky sexual behaviors and uncover inequities in access to preventive services, thereby informing more equitable and region-specific health policies and interventions.

### Conceptual framework

This conceptual framework presents a hierarchical structure in which community level factors influence individual-level factors, which in turn determines risky sexual behavior. At the top, community level factors such as residence, sub-region, impact individual-level factors, which are categorized into sociodemographic and economic factors, and behavioral factors.

The interaction between sociodemographic and economic factors and behavioral factors is expressed with a dashed arrow, indicating a weak association (Supplementary information 1).

## Methods and materials

### Study area

This study was a secondary analysis of DHS data conducted in sub-Saharan Africa countries. We included all sub-Saharan countries which incorporated risky sexual behavior assessment in their national DHS (Supplementary information 2).

### Study design and period

These surveys used a community based cross sectional study which was conducted between 2015- 2024 in Sub-Saharan Africa countries.

### Data source, sampling procedure and population

The DHS used a community-based cross-sectional study design. The study was conducted using nationally representative Demographic and Health Survey data between 2015 and 2024 in 27 SSA countries. The datasets of 27 sub-Saharan African countries can be found on the DHS website, www.dhsprogram.com [19]. The DHS sampling strategy relies on a stratified two stage probability sampling utilizing the most recent census national frames. For the first round of sampling, Enumeration Areas (EAs) were used as sampling units. The second stage of sampling was selecting a fixed number of Households (HHs), around 25–30 households per PSU, in several EAs. Each DHS report on the Measure DHS website included a comprehensive sampling technique (www.dhsprogram.com) [20].

The source population consisted of all sexually active men and women in Sub-Saharan Africa, serving as the source population from which we ultimately inferred the findings. The study population consisted of All sexually active men and women living in selected in enumeration areas of those 27 Sub-Saharan Africa countries. Regarding eligibility, the inclusion criteria consisted all men and women who have engaged in sexual activity living in selected enumeration areas of those 27 sub-Saharan Africa countries (which incorporate multiple sexual partners, inconsistent condom use in their DHS during the data collection period of the survey). And those countries with no individual and men record data for the outcome variable and survey reports before the year 2015 were excluded. Those living in selected areas with missing coordinate files were also excluded from the study.

Weighted values using the primary sampling unit or cluster variable, the stratification variable, and the weight variable. were computed to restore the representativeness of the sample data. Finally, this study comprised a total weighted sample of 428,909 (Supplementary information 3).

### Study variables

#### Dependent variable

The dependent variable for this study was risky sexual behavior (Yes/No), a derived composite binary outcome constructed from DHS based on self- reported sexual history indicators from the 12 months preceding the survey. For spatial analysis, individual level risky sexual behavior responses were aggregated within each DHS cluster to calculate a weighted cluster level prevalence. This cluster level prevalence was subsequently used as the outcome variable in spatial analysis.

#### Independent variables

##### Socio-demographic and economic factors

Age, sex, media exposure, wealth status, educational status, employment status.

##### Behavioral and clinical factors

HIV test status, age at first sex.

##### Community level factors

Residence, sub region of sub-Saharan Africa.

### Operational definitions

#### Sexually active

In this study, a respondent was considered sexually active if they reported having had sexual intercourse within the 12 months preceding the survey. Respondents who reported having had sex within the last 12 months were coded as “Yes” = 1. Respondents who reported having had sex more than 12 months ago or never had sex were coded as “No” = 0) [16].

#### Higher risk intercourse

Is defined as sexual intercourse within the last 12 months preceding the survey with a partner who was neither their spouse nor lived with them. It was coded as “Yes” if the respondent reported that their most recent, next to most recent or third to most recent sexual partner was not a spouse or cohabiting partner. Otherwise, coded as No” [21].

#### Multiple sexual partners

Participants were asked in the last 12 months; with how many different people have you had sexual intercourse? Those who reported two or more sexual partners (≥ 2) in the past 12 months were categorized as “Yes” = 1 and Those who reported zero or one partner (0–1) were categorized as “No” = 0 [21].

#### Risky sexual behavior

**Fig. 1:**
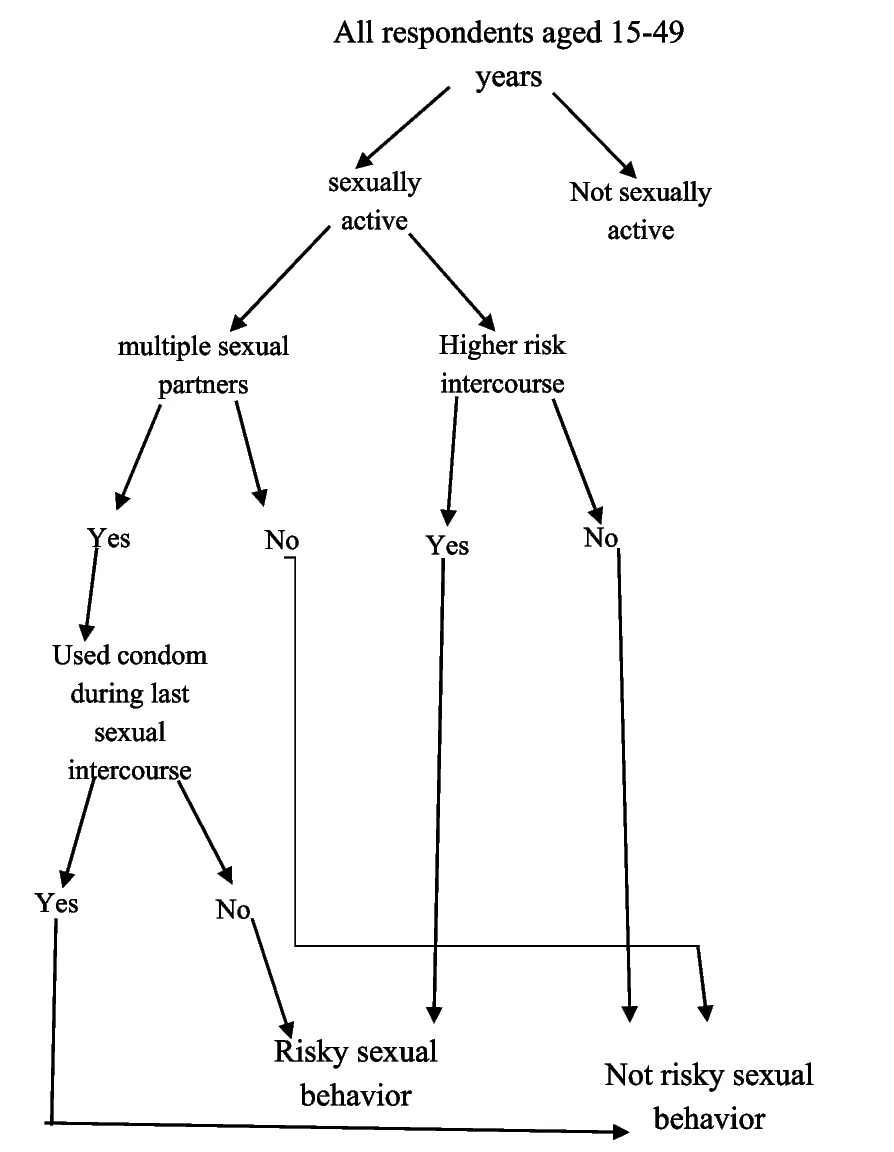
Flowchart of the operational definition and classification of the dependent variable risky sexual behavior among sexually active respondents aged 15–49

Defined as having multiple sexual partners in the 12 months and last non-use of condom among individuals with MSP or who had higher risk intercourse in the past 12 months with a person who was neither their spouse nor lived with them, which was recoded as Yes/No [21, 22] (Fig. 1).

### Data management and analysis

#### Data management

We used Stata 17 software and MS-excel 2019 for data extraction, cleaning and data management. All descriptive and analytical analysis was done using Stata 17. First, we performed exploratory data analysis to identify outliers, missing data and data distribution. We weighted our sample before any statistical analysis using weighting variable V005 and MV005 to consider unequal selection probability of samples according to DHS guideline. Missing data was managed according to DHS missing data guideline.

#### Pooled prevalence

First, the pooled prevalence of risky sexual behavior was estimated across the study countries. The weighted prevalence was calculated at the cluster level. Survey weights provided in the DHS datasets was applied in Stata, and the weighted estimates were merged with the spatial coordinates of clusters for subsequent mapping and spatial analysis.

It offers a summary estimate that accounts for variations across different populations or settings, enhancing the precision and generalizability of the findings [23, 24]. The pooled prevalence provides policymakers and healthcare providers with critical insights into the coverage gaps and informs targeted interventions to reduce risky sexual behaviors.

#### Assessment of heterogeneity

Statistical heterogeneity between studies was evaluated using τ^2^ statistic, with significance tested using Cochran’s Q test. Potential sources of heterogeneity can be considered at three levels: methodological heterogeneity includes differences in sample size, study design and measurement approaches across surveys. Clinical heterogeneity reflects differences in study populations including age distribution, sex, and regional or country specific contexts. Analytical heterogeneity refers to variations in statistical methods and effect measures to explore these potential sources, subgroup analyses were conducted by region and survey year.

#### Spatial analysis

We employed multiple spatial analysis techniques to examine the geographical variation of risky sexual behavior among sexually active men and women aged 15–49 in 27 Sub-Saharan African countries using recent DHS data. Since DHS data is geo-referenced at the cluster level, each cluster represents a single geographical point, making it the appropriate unit of spatial analysis. Because spatial analyses were conducted at the cluster level individual risky sexual behavior responses were aggregated within each cluster. After applying DHS sampling units (V005 and MV005), a weighted prevalence of risky sexual behavior was calculated for each cluster, ranging from 0 to 1. These cluster level prevalence estimates were then used as the dependent variable in the spatial regression models.

ArcGIS 10.7.1 was used for visualization, exploration, and map creation, while Stata was used for statistical analysis. The United Nations geoscheme database provides the African district delineation shape file and the coordinate points for each country was extracted in shape file format for DHS survey from the DHS office upon request. The weighted risky sexual behavior for each cluster in each survey was also calculated using Microsoft excel. After extracting the coordinates from the final survey data in ArcGIS software it was exported to STATA, then after dropping zero coordinates and no proportion records of enumeration areas it was merged with the calculated proportion for the final appended data. The coordinate projection was defined by using the World Geodetic System (WGS) 1984, and Africa Albers Equal Area conic. With regard to the analysis, spatial statistical tools were applied to analyze the data in each survey year [25].

#### Spatial autocorrelation

To determine whether risky sexual behavior is randomly distributed across geographic locations or exhibits spatial clustering, we conducted a Global Moran’s I test. Moran’s I measures spatial autocorrelation by assessing whether neighboring clusters have similar prevalence levels. A significantly positive Moran’s I would indicate spatial clustering (high-risk areas group together), while a negative value suggests dispersion (low-risk and high-risk areas are spread apart). A non-significant Moran’s I would indicate a random distribution of risky sexual behavior, suggesting no spatial dependence. The Moran’s I (*P*-value < 0.05) indicate the presence of spatial autocorrelation. Z-score was computed to determine the statistical significance of clustering, and the *p*-value was computed for the significance [26]. For local spatial analysis neighborhood relationships were defined using a default row- standardized inverse distance matrix with local pseudo *p*- values estimated by 499 conditional random permutations [27, 28].

#### Cluster and outlier analysis

It was done with the local indicator of spatial association (LISA) which identifies specific geographic locations with significantly high or low prevalence of risky sexual behavior. In addition to the local spatial autocorrelation, it was used for the determination of outlier clusters. This helps detect hot spots (clusters of high-risk behavior) and cold spots (clusters of low-risk behavior) within each country. The results were visualized using LISA cluster maps, which categorize clusters into four groups: high-high (hotspots), low-low (cold spots), high-low (outliers), and low–high (outliers) The high–high and low–low locations (positive local spatial autocorrelation) are referred to as spatial clusters, while low–high and high–low locations (negative local spatial autocorrelation) are referred to as spatial outliers [29]. Neighborhood relationships were defined using a default row- standardized inverse distance matrix, with local pseudo *p*-values estimated via 499 conditional random permutations. These maps were generated in ArcGIS and were instrumental in identifying priority areas for intervention.

#### Hotspot analysis

The local Getis-Ord statistic (Gi*), also known as hotspot analysis, was used to provide further evidence that point out the intensity and stability of core hotspot or cold spot clusters of Risky sexual behavior in the entire Sub-Saharan Africa [30]. The Getis-Ord Gi* statistic served as an indicator for local autocorrelation, which measures how spatial autocorrelation show a discrepancy locally over the study location and then computes a statistic for each point data [31].

The degree of clustering and its statistical significance were estimated based on a confidence level, according to the Z-scores. If the Z (Gi*) score is positive and significant, it shows that one district and its neighboring regions have a relatively high frequency of Risky sexual behavior, which is a hotspot (a spatial cluster of high data values); on the contrary, if the Z (Gi*) score is negative and significant, it indicates a cold-spot (a spatial cluster of low data values) [32].

#### Spatial interpolation

The spatial interpolation technique was used to predict risky sexual behavior in the un-sampled areas based on sampled enumeration areas. There are different deterministic and geo-statistical interpolation methods. In the middle of those methods, ordinary Kriging and empirical Bayesian Kriging were considered the best method since it incorporates the spatial autocorrelation and it statistically optimizes the weight [33]. The spatial interpolation method which is with small mean square error and residual value were used for this study for predictions risky sexual behavior in unobserved areas of selected sub-Saharan countries.

#### Spatial scan analysis

Spatial scan analysis was performed using Sat Scan version 10.1 software. It was employed with the Bernoulli model to identify the geographical location of a statistically significant cluster with a high proportion of risky sexual behavior. For the Bernoulli model, cases were men and women with risky sexual behavior, and controls were men and women with no risky sexual behavior. The default 50% population was used to detect primary and secondary windows of clusters. The log-likelihood ratio and *p*-value of clusters identified based on the 999 Monte Carlo replication.

#### Non-stationarity assumptions

We assessed spatial non-stationarity before proceeding with regression analysis, which occurs when the relationships between predictors and risky sexual behavior vary across locations. To assess the extent of clustering, we computed the Intra-Class Correlation (ICC), which measures the proportion of variance in risky sexual behavior attributed to clustering.

#### Spatial regression

To identify the appropriate explanatory variables for spatial regression analysis, an exploratory regression analysis was conducted. Exploratory regression systematically tests all possible combinations of explanatory variables to determine which models satisfy key statistical assumptions, including statistical significance of coefficients, absence of multicollinearity, normality of residuals, and lack of spatial autocorrelation. This process ensures that only well-specified models are considered for further analysis [34].

Once an optimal model was identified, an Ordinary Least Squares (OLS) regression was performed to establish a global relationship between the dependent variable (Risky sexual behavior) and the explanatory variables. OLS is widely used in spatial analysis as it provides a global regression model representing the entire dataset using a single equation. However, it assumes that relationships between predictors and the dependent variable remain constant across the study area, which may not always hold in spatial data analysis [35].

To evaluate the performance of the OLS model, several diagnostics were conducted: Model Significance: The overall significance of the model will be assessed using the F-statistic and its associated *p*-value. A *p*-value < 0.05 indicates that the model is statistically significant and that at least one explanatory variable has a significant relationship with the dependent variable [36]. Stationarity: The Jarque–Bera statistic was used to test the assumption of residual normality, where a significant *p*-value indicates that the error terms deviate from a normal distribution, separately, spatial non stationarity was tested using the Koenker BP statistic, where a *P*-value < 0.05 indicates that the relationship between the independent variable and dependent variable was geographically violating stationarity.

Additionally, a variance inflation factor (VIF) greater than 10 was used to identify multicollinearity among the explanatory variables, ensuring that only non-collinear variables are retained in the model [37].

#### Geographically Weighted Regression (GWR)

Geographically Weighted Regression (GWR) exploratory analysis was performed to examine whether risk factors for risky sexual behavior have varying effects in different geographic areas. Since spatial non-stationarity was detected, classical regression models were not appropriate, and spatial regression models were required to account for these variations.

The analysis began with an Ordinary Least Squares (OLS) regression model, followed by a check for spatial autocorrelation in the residuals using Moran’s I since significant spatial dependence existed, spatial regression models were required. Geographically Weighted Regression (GWR) was then applied to model the spatially varying relationships between individual and community level factors and risky sexual behavior. To account for potential spatial non-stationarity in the relationships, a geographically weighted regression (GWR) was employed. Unlike OLS, GWR generates location-specific regression coefficients, allowing for variations in relationships across different geographic units. This approach was particularly useful in identifying spatial heterogeneity and improving model accuracy by addressing localized patterns in the data. In the GWR analysis, a *p*-value < 0.05 was considered statistically significant [38].

Ordinary Least Squares (OLS) regression was first fitted to assess the global relationship between explanatory variables and risky sexual behavior. Model diagnostics indicated significant spatial non-stationarity (Koenker BP test, *p* < 0.05), suggesting that the relationships between predictors and risky sexual behavior varied across geographic locations. In addition, the residuals deviated from normality (Jarque–Bera test, *p* < 0.05), indicating that a single global model may not adequately represent spatially varying relationships. Therefore, Geographically Weighted Regression (GWR) was employed to account for local spatial heterogeneity by estimating location-specific coefficients.

For the GWR configuration, an adaptive bi-square kernel function was employed to select the local neighborhood size, allowing the bandwidth to vary dynamically based on the density of the DHS clusters. The optimal bandwidth was determined by minimizing the corrected Akaike Information Criterion (AICc). To validate the model’s performance post-estimation, residual diagnostics were conducted by running a Global Moran’s I test on the standardized residuals to verify that spatial autocorrelation was successfully eliminated and that the model errors were randomly distributed [38].

## Result

### Socio-demographic and economic characteristics of participants

A total of 428,909 participants were included in this study. The majority of participants were female (70.4%). Regarding education, 33.28% of participants completed secondary education. Most participants were in the age group of 25–29 years (19.24%). About 57.56% of participants reported having their first sexual intercourse before the age of 18. Around 70.58% of the participants were currently working, while others were not. About 44% of the total participants were from rich households.

About 75.93% of the participants were married. In terms of media exposure, 56.1% participants were exposed to media and majority (65.32%) of participants reported having ever been tested for HIV (Supplementary information 4).

### Pooled prevalence of risky sexual behavior

**Fig. 2:**
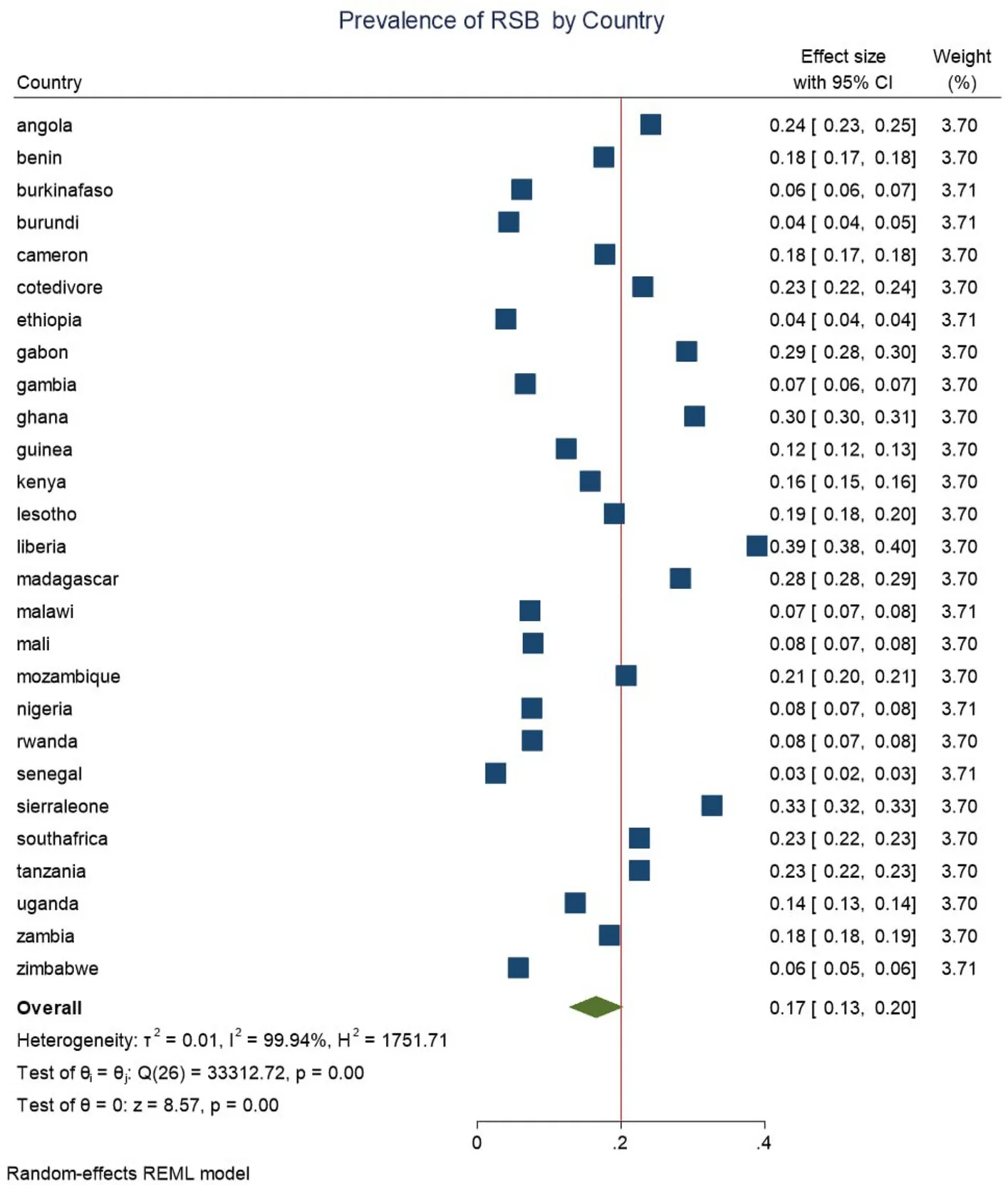
Forest plot of overall prevalence of risky sexual behavior in Sub Saharan Africa countries from 2015–2024

The pooled prevalence of risky sexual behavior across 27 Sub-Saharan African countries was estimated at 17% (95% CI: 13–20%). Prevalence estimates varied considerably across countries, ranging from 3% in Senegal to 39% in Liberia. There was significant heterogeneity among the countries (I^2^ = 99.94%, τ^2^ = 0.01, *p* < 0.001), indicating that most of the variability in prevalence estimates was due to real differences between countries rather than chance (Fig. 2).

To explore this heterogeneity, a subgroup analysis by region and survey year was conducted. The overall pooled prevalence of risky sexual behavior in Sub-Saharan Africa was 17% (95% CI: 13%–20%), with significant heterogeneity across regions (I^2^ = 99.92%, *p* < 0.001). The highest prevalence was observed in Central Africa (24%), followed by Southern (16%) and Western Africa (17%), while Eastern Africa had the lowest (14%). Country-level estimates ranged widely, from 3% in Senegal to 39% in Liberia, highlighting substantial regional variation (Supplementary information 5).

Countries surveyed between 2015 and 2020 had a pooled prevalence of 14% (95% CI: 10–18%, I^2^ = 99.94%, τ^2^ = 0.01), while those from year 2021 and above had a higher prevalence of 21% (95% CI: 14–27%, I^2^ = 99.94%, τ^2^ = 0.01). However, the difference between the groups was not statistically significant, indicating that survey year alone may not explain the observed variation (Supplementary information 6).

### Spatial variation of risky sexual behavior in sub- Saharan Africa

Figure below showed that the spatial variation of Risky sexual behavior was found in SSA. For instance, the red dotes indicate areas with a higher proportion of risky sexual behavior and the green dots indicate areas with a lower proportion of risky sexual behavior (Supplementary information 7).

### Global spatial autocorrelation (Moran’s I) analysis

The spatial pattern of risky sexual behavior in sub-Saharan Africa was clustered across enumeration areas. The global spatial autocorrelation analysis depicted that there was a significant clustered pattern of risky sexual behavior in the enumeration areas of sub-Saharan Africa (Global Moran’s *I* = 0.42, Z-score = 131.294738, *p*-value < 0.01). This output showed that the Z-score was positive, with a highly significant *p*-value that would be interpreted as 99% confidence for the clustering of risky sexual behavior across the enumeration in Sub Saharan Africa. The figures below depict the clustered patterns (on the right side) with high rates of Risky sexual behavior across clusters in sub–Saharan Africa. The bright red and blue colors (to the right and left sides) indicated an increased significance level for which the likelihood of clustered patterns occurring by random chance was less than 1% (Supplementary information 8).

### Cluster and outlier (Anselin Local Moran’s *I*) analysis

**Fig. 3:**
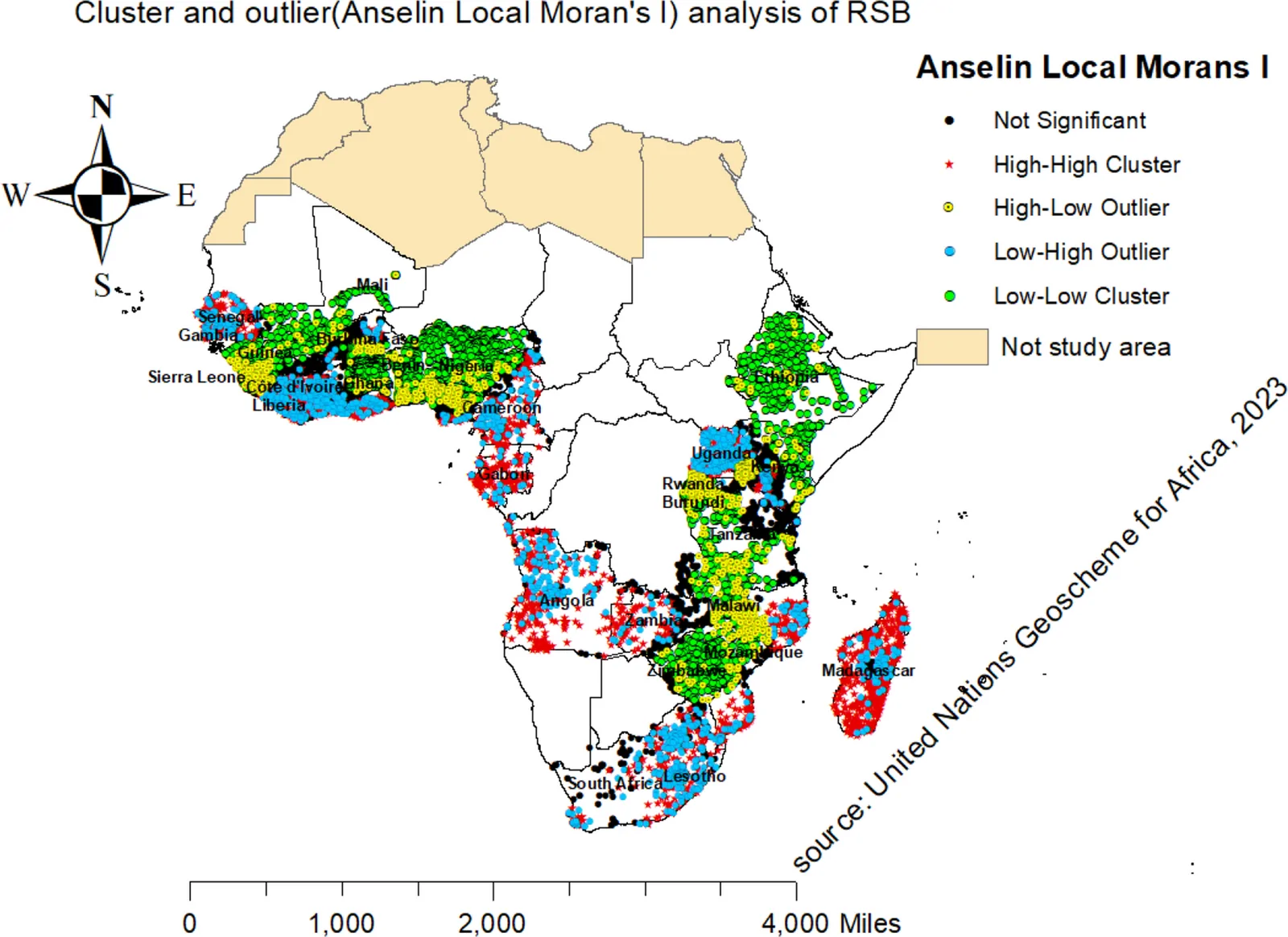
Local cluster and outlier (Anselin Local Moran’s I) analysis

Anselin Local Moran’s I analysis showed significant high-high clusters of risky sexual behavior in southern Angola, central and southern Mozambique, South Africa, Lesotho, and parts of Zimbabwe, while low-low clusters of risky sexual behavior were observed in southern Ethiopia, northern and western Kenya, Rwanda, Burundi, Malawi, Uganda, Tanzania, and parts of Zambia (Fig. 3).

### Hotspots (Getis-Ord Gi*) analysis of risky sexual behavior

The areas with high risky sexual behavior were detected in southern Angola, central and southern Mozambique, Lesotho, South Africa, southern Zimbabwe, and the coastal areas of Côte d’Ivoire, Liberia, and Sierra Leone. Whereas areas with low risky sexual behavior were identified in Ethiopia, northern and western Kenya, Uganda, Rwanda, Burundi, Tanzania, northern Malawi, and parts of Burkina Faso, Ghana, Togo, and Benin (Supplementary information 9).

### Spatial interpolation

**Fig. 4:**
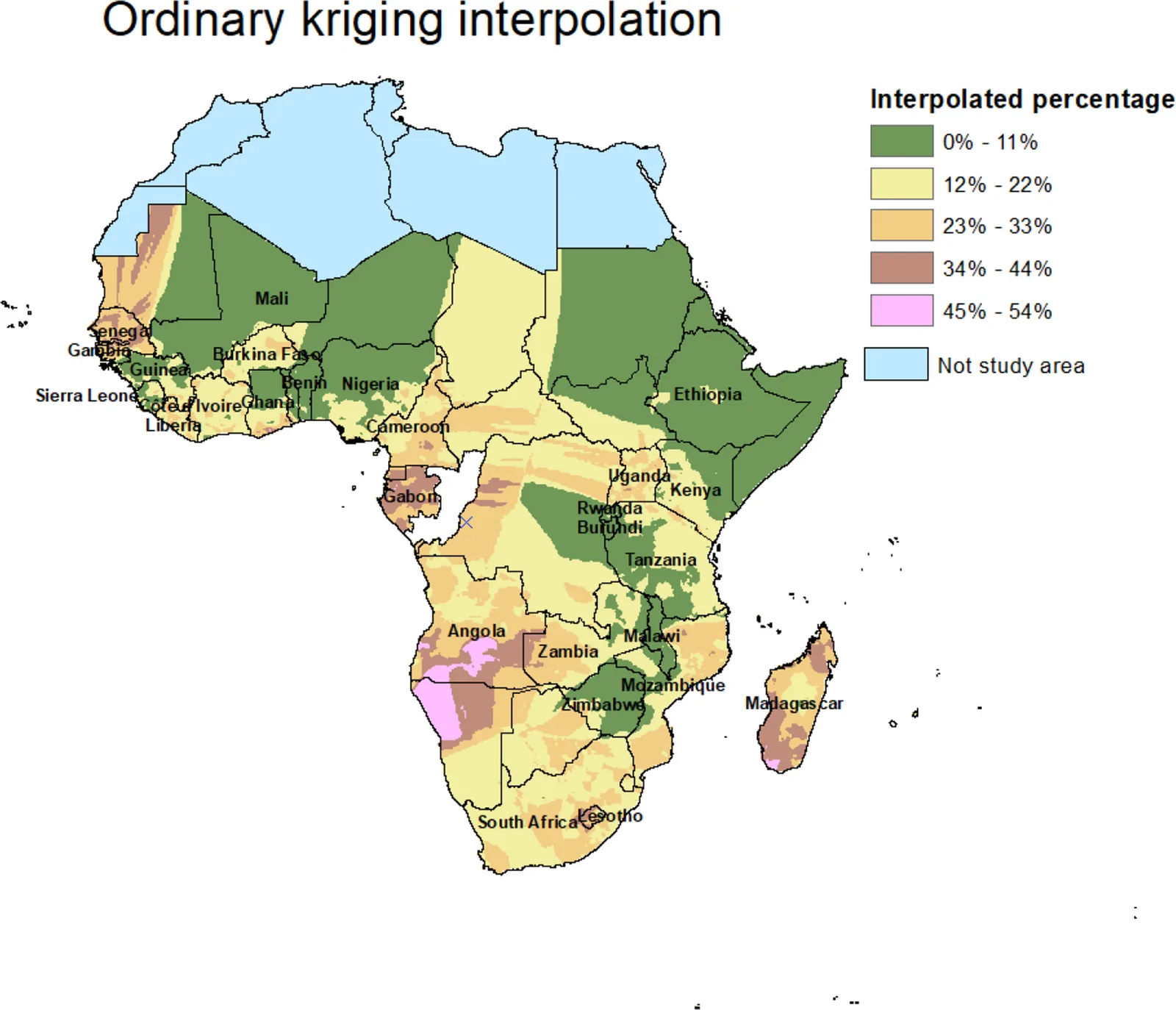
Ordinary kriging interpolation of risky sexual behavior in SSA

As shown in Fig. 4, ordinary kriging interpolation was used to map the continuous spatial distribution of risky sexual behavior across sub- Saharan Africa. The highest predicted risk categories are concentrated in southern Africa across southwestern Angola, Lesotho and Madagascar. Conversely low prevalence zones cover wide areas of east Africa including Ethiopia, Kenya and Uganda moderate risk zones include Senegal, Guinea, Sierra Leone, Liberia, and Côte d’Ivoire (Fig. 4).

### Sat scan cluster analysis of risky sexual behavior

Based on Sat Scan cluster analysis, out of 426,605 total individuals, 68,212 (16.0%) individuals had risky sexual behavior in the selected enumeration areas. A total of 5589 significant clusters were detected in the 15 most likely clusters (Supplementary information 10).

The first most likely big cluster encompassed mainly Senegal, Angola, parts of Mozambique, Zambia, Gabon, Madagascar, Lesotho and some part of Cameroon. It was found at (15.988095 N, 16.492659 W)/506.78 km radius. In the primary most likely cluster, 7076 (33%) individuals had Risky sexual behavior, (LLR = 2057.026823; *P*-value < 0.01) (Supplementary information 11).

The second most likely Sat Scan cluster covered mainly Uganda and parts of Nigeria which was located at (6.948997 S, 15.233172 E)/1561.35 km radius. Clusters in the second SaT Scan window were 1.71 times more likely to have Risky sexual behavior compared with those outside this window (RR = 1.54 LLR = 1425.880820; *P*-value < 0.01).

The third most likely cluster encompassed mainly Cote d’ivoire, Ghana, and Liberia. This SaT Scan cluster was found at (29.462799 S, 27.686337 E)/120.25 km radius, and in this cluster, 3946 (36.4%) had Risky sexual behavior. Clusters in this window were 2.34 times higher outside this window to have risky sexual behavior (RR = 2.32, LLR = 1273.784965, *P*-value < 0.01).

### Spatial regression analysis

#### Ordinary least square analysis (OLS)

Six of the OLS model assumptions were checked to evaluate whether the model was best fit or biased. All the coefficients of the explanatory variables had either a positive or negative relationship with risky sexual behavior and several were statistically significant. Redundancy or multicollinearity among explanatory variables was not observed, as all VIF values were below the recommended threshold of 7.5. The OLS model performance revealed that about 33.7% (adjusted R^2^ = 0.3369) of the variation in Risky sexual behavior was explained by the included predictors, and the model’s AICc value was 124639.097710 (supplementary information 12).

The Jarque–Bera statistic was statistically significant, indicating that the residuals deviated from a normal distribution. However, the Koenker BP statistic was also highly significant, showing a spatial non stationarity. This confirmed that the relationships between predictors and risky sexual behavior vary significantly across geographic space, indicating the presence of spatial variation in the coefficients across different enumeration areas (EAs). Therefore, fitting a geographically weighted regression model was required to account for this spatial heterogeneity and improve prediction accuracy.

The joint F-statistic and Wald Chi-squared test showed that the overall OLS model was statistically significant (*p*-value < 0.001). Because the Koenker test indicated non-stationarity, robust probabilities were used to determine the statistical significance of each coefficient. Accordingly, the proportion of individuals who are Not working (*p* < 0.001), those who are tested for HIV (*p* < 0.001), Males (*p* < 0.001), Not married (*p* < 0.001), and exposed for media (*p* < 0.001) were significantly associated with risky sexual behavior in the study population. These findings justify the relevance of the selected predictors while highlighting spatially varying effects that can be better captured using GWR analysis.

#### Geographically Weighted Regression (GWR) analysis

The local spatial regression (GWR analysis) was conducted because two of the six assumptions in the OLS regression were not met, in which both the Jarque–Bera statistic and the Koenker (BP) were significant. In the GWR analysis, model performance was improved over the OLS model regression. The adjusted R square increased from 33.7% in the OLS analysis to 56.2% in the GWR analysis, indicating that the GWR model better explained the geographical variation of Risky sexual behavior among sexually active men and women in Sub Saharan Africa. Moreover, the AICc value of 124639.0978 in the OLS model was reduced to 118073.8894 in the GWR model, which revealed a better model fit to explore Risky sexual Behavior using GWR (Table 1).

**Table 1:** Summary of geographically weighted regression (GWR) analysis of risky sexual behavior among sexually active men and women in sub-Saharan Africa

Neighbors	329583	AICc	118073.889441
Effective number	327.88	Adjusted R square	56.24%
Sigma	9.258826		

The GWR analysis demonstrated a spatially varying relationship between being not working and risky sexual behavior among sexually active men and women in Sub-Saharan Africa. The coefficients ranged from −0.702460 to 3.014586, indicating both negative and positive associations across different regions.

**Fig. 5:**
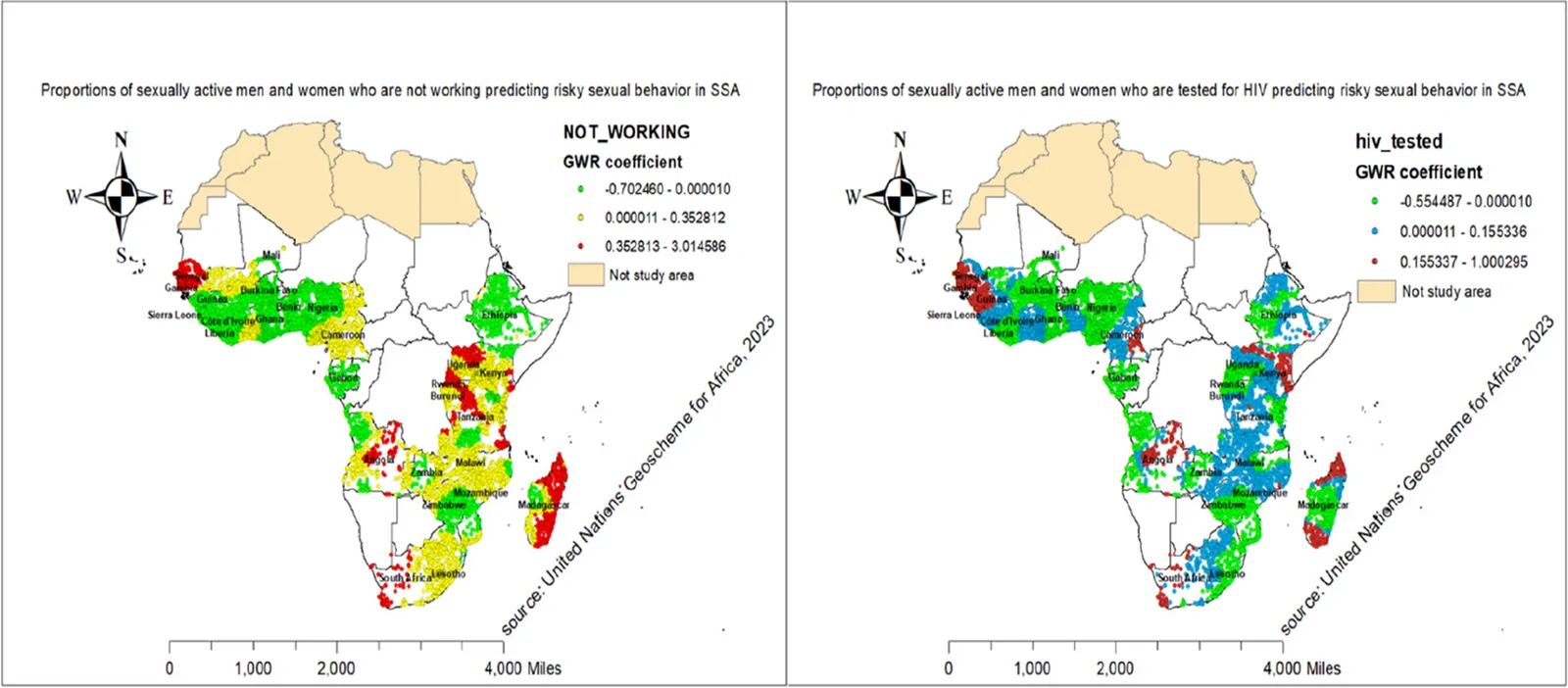
**A** The GWR coefficients of not working, **B** The GWR coefficients of HIV tested

In Uganda, Kenya, Rwanda, Burundi, Tanzania, Angola, South Africa, and Madagascar, not working was a strong and positive predictor of risky sexual behavior. In these areas, the prevalence of risky sexual behavior increased as the proportion of sexually active individuals who were currently working increased. In contrast, a strong and negative relationship was observed in Nigeria, Burkina Faso, parts of Cameroon, Ethiopia, Malawi, and Zimbabwe. In these areas, higher proportions of individuals not working were associated with lower levels of risky sexual behavior, as shown by the dark green areas with lower negative coefficients. A weak or neutral association was seen in parts of South Africa, Ghana, Côte d’Ivoire, Zambia, Mozambique, and Lesotho (Fig. 5A).

HIV testing was a strong and positive predictor of risky sexual behavior In Kenya, Uganda, South Africa, Madagascar, parts of Angola, and small areas of Cameroon and Côte d’Ivoire. In these locations, as the proportion of sexually active individuals who had been tested for HIV increased, the prevalence of risky sexual behavior also rose. In contrast, a negative association was observed in Nigeria, Mali, Burkina Faso, Benin, Togo, Gabon, parts of Angola, and Mozambique. In these areas, increases in HIV testing were associated with decreases in risky sexual behavior. A weak positive association was found in much of Ethiopia, Tanzania, Malawi, Zambia, Rwanda, Burundi, and parts of Ghana, where HIV testing had minimal or mixed influence on risky sexual behavior. These regions, represented by blue symbols, indicate areas where the relationship between testing and behavior is less pronounced or varies on a more localized scale (Fig. 5B).

Being male demonstrated a spatially varying relationship with risky sexual behavior among sexually active individuals across Sub-Saharan Africa. In some areas, it was a positive predictor, while in others it showed a negative association.

**Fig. 6:**
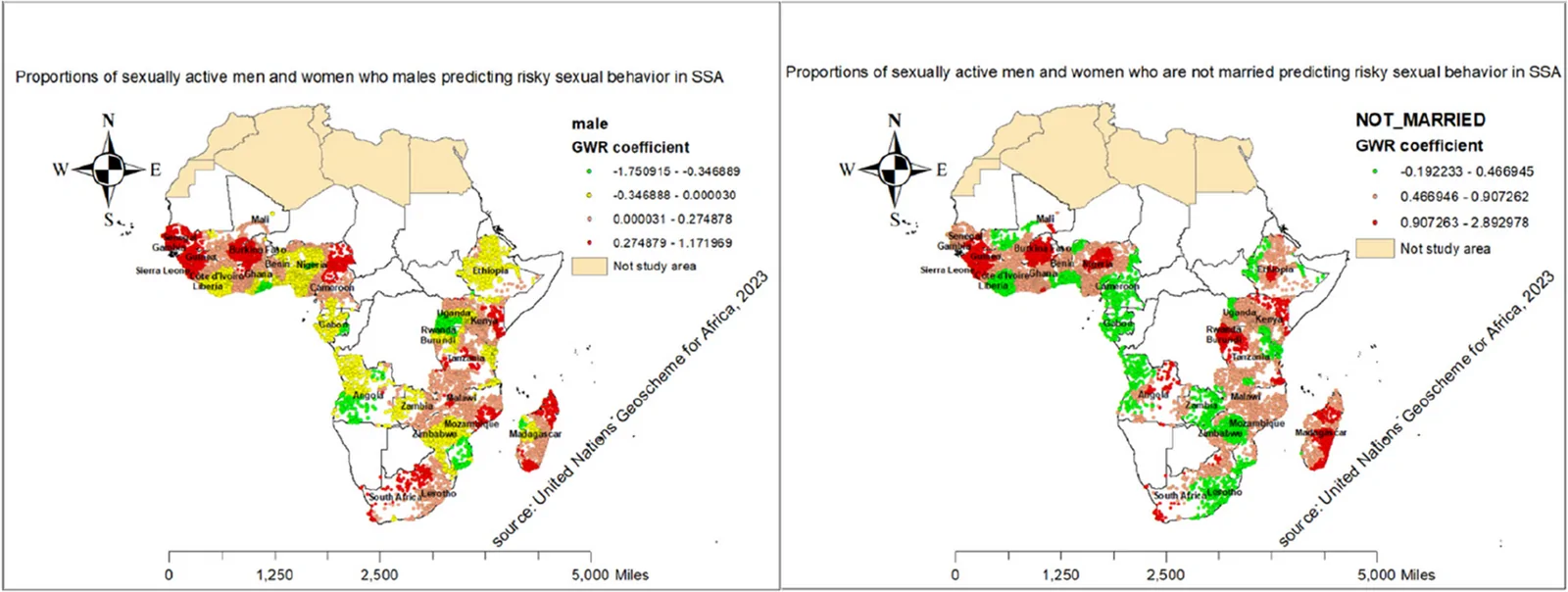
**A** The GWR coefficients of males, **B** The GWR coefficients of not married

In Senegal, Gambia, Guinea, Sierra Leone, Côte d’Ivoire, Burkina Faso, Benin, parts of Cameroon, Lesotho, South Africa, and Madagascar, being male was a strong and positive predictor of risky sexual behavior. In these regions, as the proportion of sexually active males increased, the prevalence of risky sexual behavior also increased. In contrast, a negative relationship was observed in Angola, Zambia, parts of Gabon, Zimbabwe, and small areas in Mozambique. In these areas, increases in the proportion of sexually active males were associated with decreases in risky sexual behavior. A weak association was found in much of Kenya, Uganda, Tanzania, Rwanda, Burundi, Malawi, Ethiopia, northern Mozambique, and Nigeria, where being male had a mixed or minimal influence on risky sexual behavior (Fig. 6A).

Being not married was a strong and positive predictor of risky sexual behavior in Kenya, Uganda, Rwanda, Burundi, Tanzania, Angola, South Africa, and Madagascar. In these regions, as the proportion of sexually active individuals who were not married increased, the prevalence of risky- sexual behavior also increased. In Nigeria, Burkina Faso, Cameroon, Ethiopia, Malawi, and Zimbabwe, a negative association was observed. In these areas, unmarried sexually active individuals were associated with lower levels of risky sexual behavior.

A weak relationship was observed in parts of Ghana, Côte d’Ivoire, Zambia, Mozambique, and Lesotho, where being unmarried had minimal influence on risky sexual behavior. This indicates that marital status alone may not be a strong determinant of RSB in these areas (Fig. 6B).

**Fig. 7:**
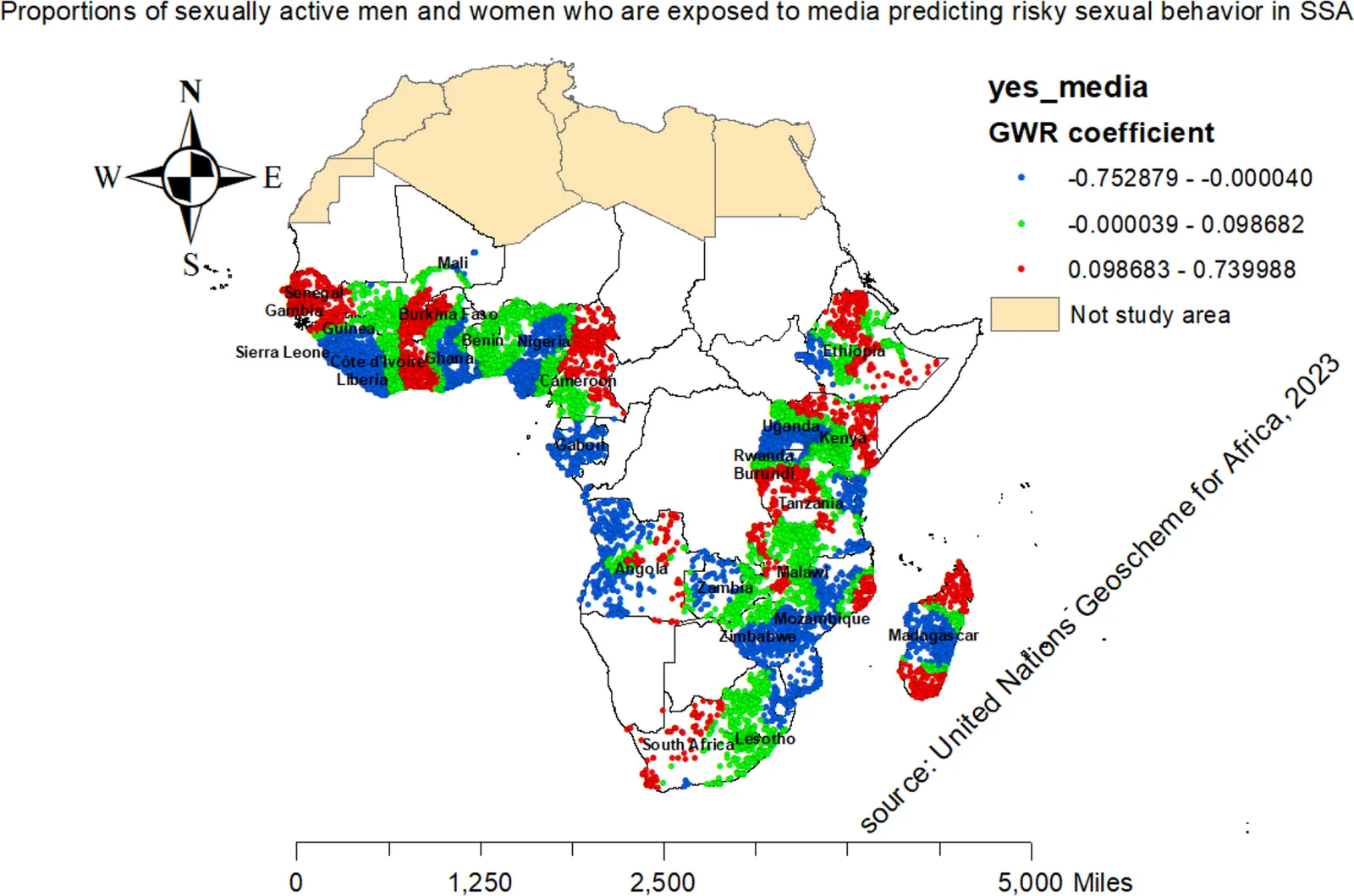
The GWR coefficients of media exposure predicting risky sexual behavior among sexually active men and women in sub- Saharan Africa, using recent DHS (2015–2024)

In Ethiopia, Kenya, Rwanda, Burundi, Madagascar, and parts of Nigeria, Cameroon, Senegal, and Gambia, media exposure was a strong and positive predictor of risky sexual behavior. As the proportion of sexually active individuals exposed to media increased in these regions, the prevalence of risky sexual behavior also increased. In contrast, media exposure was a negative predictor of risky sexual behavior in Angola, Zambia, Zimbabwe, southern Gabon, and parts of the Democratic Republic of Congo. In these areas, an increase in media exposure was associated with a decrease in risky sexual behavior. A weak association was found in parts of Ghana, Côte d’Ivoire, Burkina Faso, Malawi, Mozambique, Lesotho, and South Africa, where media exposure had minimal influence on risky sexual behavior (Fig. 7).

## Discussion

This study aimed to determine the overall prevalence, spatial variation and associated factors of risky sexual behavior in SSA using advanced statistical models. The finding of this study showed that Risky sexual behavior had significant spatial variation in SSA. Getis-Ord spatial analysis showed that hotspot, cold spot, and outlier clusters were detected using cluster outlier analysis statistically significant high-high clusters, where countries with high RSB prevalence were surrounded by others with similarly high values, as well as low-low clusters and high-low outliers.

In spatial Regression analysis, different factors were found to have a statistically significant effect on Risky Sexual Behavior. Accordingly, being male, not working currently, being exposed to media, being tested for HIV, being not married were the significant predictors of Risky sexual behavior.

In this study around 68,212 (17%, 95%CI: 13% to 20%), had risky sexual behavior in sub-Saharan Africa. this finding is higher than studies from An European survey which is 9.8% of students reported unprotected sex, one key risky behavior, with some country estimates as low as ~ 3% [39]. A global systematic review also reported a wide range of RSB across different regions, with the lowest estimates around 7.6% [40]. It is also higher than a study conducted in Sri Lanka. Prevalence of RSB in last 1 year and 3 months periods were 12.4%, (95% CI: 11.8–13.1) and 12.1%. These differences may reflect variations in cultural norms, educational interventions, or definitions of behaviors across studies [41]. But this finding is lower than many country-specific studies previously reported. For example, the prevalence of RSB was 48.3% in South Africa [42], 57.7% in Nigeria [43], and 28.16% among reproductive-age women in SSA [44]. Similarly, studies from Uganda (24.2%) and South Ethiopia (43.7% among ART attendees) [45, 46] also reported higher levels of risky sexual behavior. However, some of these differences may come from variations in target populations for instance women only, adolescents, ART clients or the specific definitions of RSB used in each study.

The spatial variation of the predictors revealed notable geographic heterogeneity in the association between socio-demographic and behavioral factors and RSB, reinforcing prior findings from Ethiopia [47], Nigeria [48], and Malawi [49] that documented spatial clustering in sexual risk behaviors. This supports the idea that regional socio-cultural practices and community-level dynamics can significantly influence sexual behavior [50].

The GWR analysis identified regions where not working was a strong and positive predictor of RSB, particularly in Uganda, Kenya, Rwanda, Burundi, Tanzania, Angola, South Africa, and Madagascar. This shows that un employment was associated with risky sexual behavior. This finding partially aligns with prior research from Ghana and Kenya, where employment status was associated with increased odds of RSB [51]. However, it contradicts some studies from Kenya where employment appeared protective, underscoring the spatial heterogeneity observed in this study. Conversely, in Nigeria, Burkina Faso, Cameroon, Ethiopia, Malawi, and Zimbabwe, not working was negatively associated with RSB. These findings may reflect regional differences in the socioeconomic implications of unemployment and varying access to opportunities or support systems. In contrast, regions like Ghana, Côte d’Ivoire, Zambia, Mozambique, and Lesotho showed weak or no association, indicating that employment status may not uniformly predict RSB across SSA.

HIV testing also showed spatial variation in its relationship with RSB. In Kenya, Uganda, South Africa, Madagascar, and parts of Angola and Cameroon, HIV testing positively predicted RSB. This aligns with findings from Senegal, which suggested that individuals testing negative for HIV may feel a reduced sense of vulnerability and engage in riskier behavior [52]. However, in Nigeria, Mali, Burkina Faso, and Mozambique, a negative relationship was observed, potentially reflecting the protective effect of HIV awareness and counseling efforts following testing in those contexts. A weak relationship in Ethiopia, Tanzania, and Malawi further highlights the varying public health messaging and intervention effectiveness across SSA.

Male sex showed considerable spatial variability in its influence on RSB. Being male was a strong and positive predictor in Senegal, Gambia, Guinea, Côte d’Ivoire, Burkina Faso, Benin, parts of Cameroon, and Madagascar. This corresponds with multiple studies across SSA reporting higher RSB among men [53–55]. Conversely, in Angola, Zambia, and Zimbabwe, being male was negatively associated with RSB, while in countries like Kenya, Uganda, and Nigeria, the relationship was weak or mixed. These regional contrasts may be due to differences in cultural norms, masculinity ideals, or HIV prevention programs targeting men.

Marital status, particularly being not married, was found to be a strong predictor of RSB in Eastern and Southern SSA, including Kenya, Uganda, and Madagascar. This aligns with studies in Zambia and SSA more broadly, which found that unmarried individuals were more likely to engage in risky sexual practices [11, 56]. In contrast, in parts of Nigeria, Cameroon, and Malawi, not being married was negatively associated with RSB, suggesting contextual differences in sexual networks and norms surrounding premarital sex. Weak associations in countries such as Ghana and Zambia imply that marital status alone may not fully explain RSB patterns and should be considered alongside other cultural and behavioral factors.

Media exposure showed spatially varying relationships with RSB. In Ethiopia, Kenya, Rwanda, and Nigeria, it was a strong positive predictor, consistent with findings from Ethiopia and Nigeria indicating that exposure to media is often strongly linked to risky sexual behaviors through indirect messaging [43, 57]. However, in Angola, Zambia, and parts of DRC, media exposure was negatively associated with RSB, potentially reflecting the effectiveness of targeted public health campaigns. In areas with weak associations, such as Ghana and Mozambique, the impact of media on behavior may be diluted due to limited access or content irrelevance.

Contextually, these patterns are heavily influenced by local policy execution; while STI screenings and condoms are theoretically free in public facilities, unmarried populations face severe provider-driven stigma and frequent resource stockouts [58, 59]. Furthermore, unmarried individuals face severe provider driven stigma and lack of youth friendly spaces at these facilities which actively inhibits them from obtaining free preventive tools and exacerbates localized risky sexual practices [60].

The considerable spatial heterogeneity revealed across sub- Saharan Africa demonstrates that a uniform approach to reducing risky sexual behavior will be highly inefficient. Instead, national ministries of Health must translate these distinct spatial clusters and GWR outputs into integrated, country specific priority intervention frameworks. For instance, in east and southern nations such as Kenya, Uganda and Madagascar where being unmarried and not working emerged as strong positive predictors of RSB, policy frameworks must combine economic empowerment with localized, youth-friendly spaces that remove provider-driven stigma. Conversely, in West African contexts like Senegal and Guinea, where male sex was highly a predictor, public health campaigns must pivot toward gender specific behavior change strategies.

## Strength and limitation of the study

The analysis of this study was based on nationally representative and most recent DHS data, which was collected by standardized and validated data collection instruments. Besides, the use of Geographic Information System (GIS) and SaT Scan statistical tests helped to detect similar and statistically significant high-risk clusters of Risky sexual behavior. Moreover, a major strength of this study is the use of GWR, which allows for the identification of spatially varying relationships, unlike traditional regression models that assume uniformity across space. This approach helps to identify context-specific drivers of RSB that are otherwise masked in national-level analyses. Additionally, this study covers both men and women and includes a wide age range, offering a more inclusive perspective.

However, the study also has limitations. important behavioral and contextual factors such as substance use, peer pressure, and detailed socio-cultural norms were not captured in the dataset. Also, the use of self-reported sexual behavior may be prone to social desirability and recall biases and since this study is from a 10-year dataset this behavioral change might be underestimated. Additionally, this study utilizes georeferenced cluster coordinates that are intentionally displaced to ensure confidentiality and this displacement introduces a minor spatial uncertainty that may cause slight localized misclassifications during hotspot detection and local spatial estimations.

## Conclusion and recommendations

This study found that risky sexual behavior (RSB) remains a significant public health concern in Sub-Saharan Africa, with an overall prevalence of 17% and considerable spatial variation across countries. The use of advanced spatial analysis techniques revealed geographic clustering of high and low prevalence areas, and the Geographically Weighted Regression (GWR) analysis showed that predictors such as sex, employment status, media exposure, HIV testing, and marital status had location-dependent effect on risky sexual behavior. The findings highlight the importance of working beyond generalized regional strategies towards geographically oriented interventions that aim at the socio-cultural and behavioral contexts of each country. According to the study findings, Policymakers should promote geographically focused strategies by incorporating spatial data into national health planning and addressing structural factors such as unemployment, which was linked to risky sexual behavior. Public health practitioners are advised to strengthen post-HIV test counseling and develop culturally sensitive interventions that consider variations in gender, marital status, and media exposure. NGOs and community organizations should apply behavior change communication campaigns and community engagement initiatives that are based on local social and cultural settings. Media and communication agencies are encouraged to provide responsible, relevant messaging that reinforce positive sexual health behaviors and counters harmful norms. Finally, researchers should continue using advanced spatial models to gain a further insight into risky sexual behavior and assess the effect of geographically targeted interventions. And also apply more recent datasets to capture temporal changes of behaviors.

## Supplementary Information

Supplementary Material 1.

## Data Availability

The dataset used for this study is publicly available at the MEASURE DHS program website (https://www.dhsprogram.com/data/).

## Abbreviations

AIDS: Acquired Immunodeficiency Syndrome
ART: Antiretroviral Therapy
CDC: Centers for Disease Control and Prevention
DHS: Demographic Health Survey
EA: Enumeration Area
EDHS: Ethiopian Demographic and Health Survey
ETB: Ethiopian Birr
GB: Gigabyte
GPS: Global Positioning System
HRSB: High Risk Sexual Behavior
HH: House Hold
ICC: Intra-Class Correlation
LISA: Local Indicators of Spatial Association
MOR: Median Odds Ratio
MSP: Multiple Sexual Partners
NGOs: Non-Governmental Organizations
PCV: Proportional Change in Variance
PSU: Primary Sampling Unit
RAM: Random Access Memory
RSB: Risky Sexual Behavior
SDG: Sustainable Development Goal
SSA: Sub-Saharan Africa
STD: Sexually Transmitted Disease
STI: Sexually Transmitted Infections
VIF: Variance Inflation Factor
WHO: World Health Organization
