# Supplementary material for: Pooled prevalence, spatial variation and associated factors of risky sexual behavior among sexually active men and women aged 15–49 years in sub-Saharan Africa, 2015–2024: evidence from Demographic and Health Survey
Source: BMC Public Health. 2026 Jul 15;26:2925. doi: 10.1186/s12889-026-28529-0 (PMC13651065; doi:10.1186/s12889-026-28529-0)
Supplement: Supplementary file 1 [file 12889_2026_28529_MOESM1_ESM.pdf]

Supplementary information 1

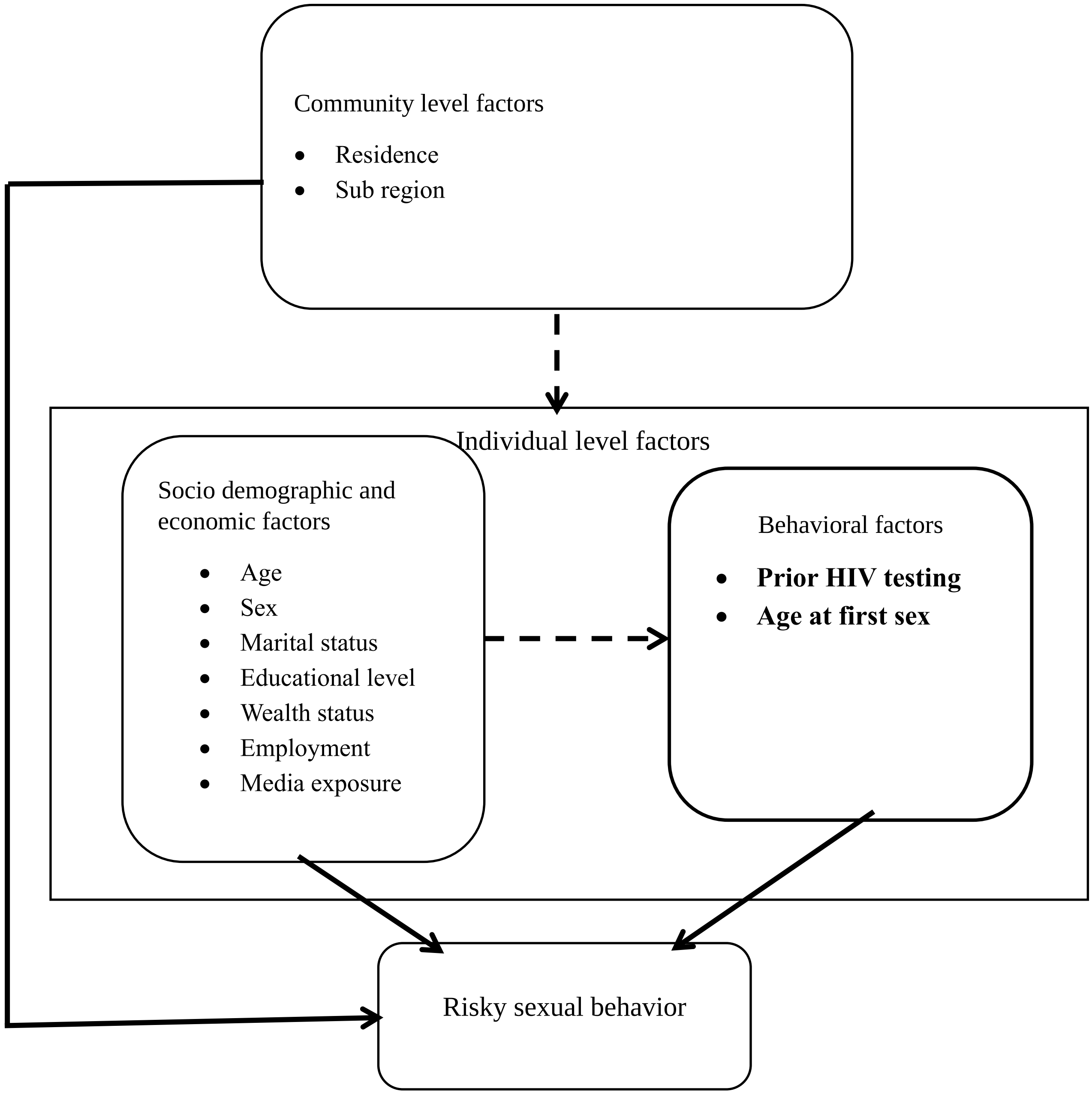

Figure: Conceptual framework on associated factors of risky sexual behavior among sexually active adults in sub- Saharan Africa

Supplementary information 2

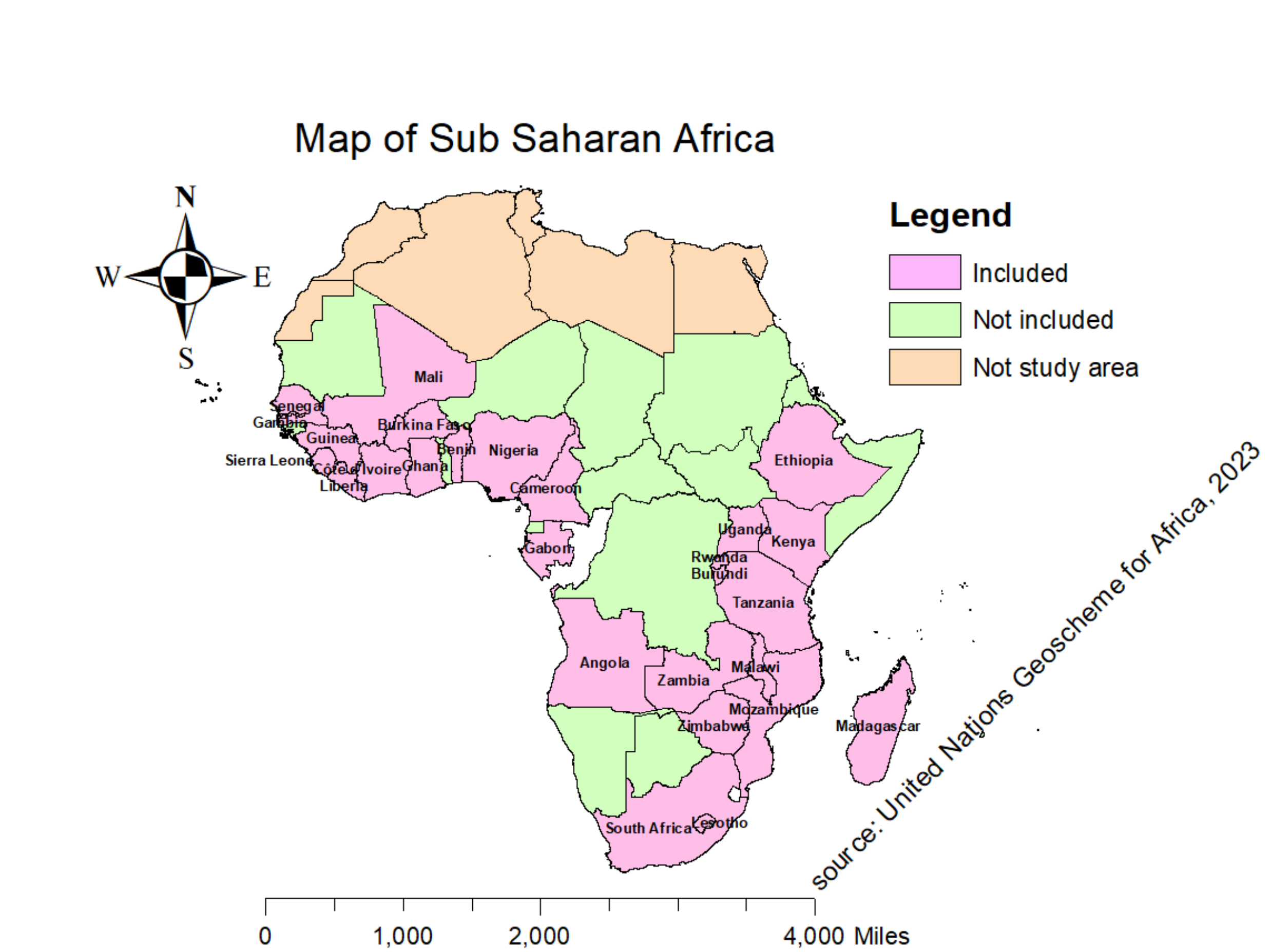

Figure: Study area of pooled prevalence, spatial variation and associated factors of risky sexual behavior among sexually active men and women in SSA (2015-2024)

Supplementary information 3

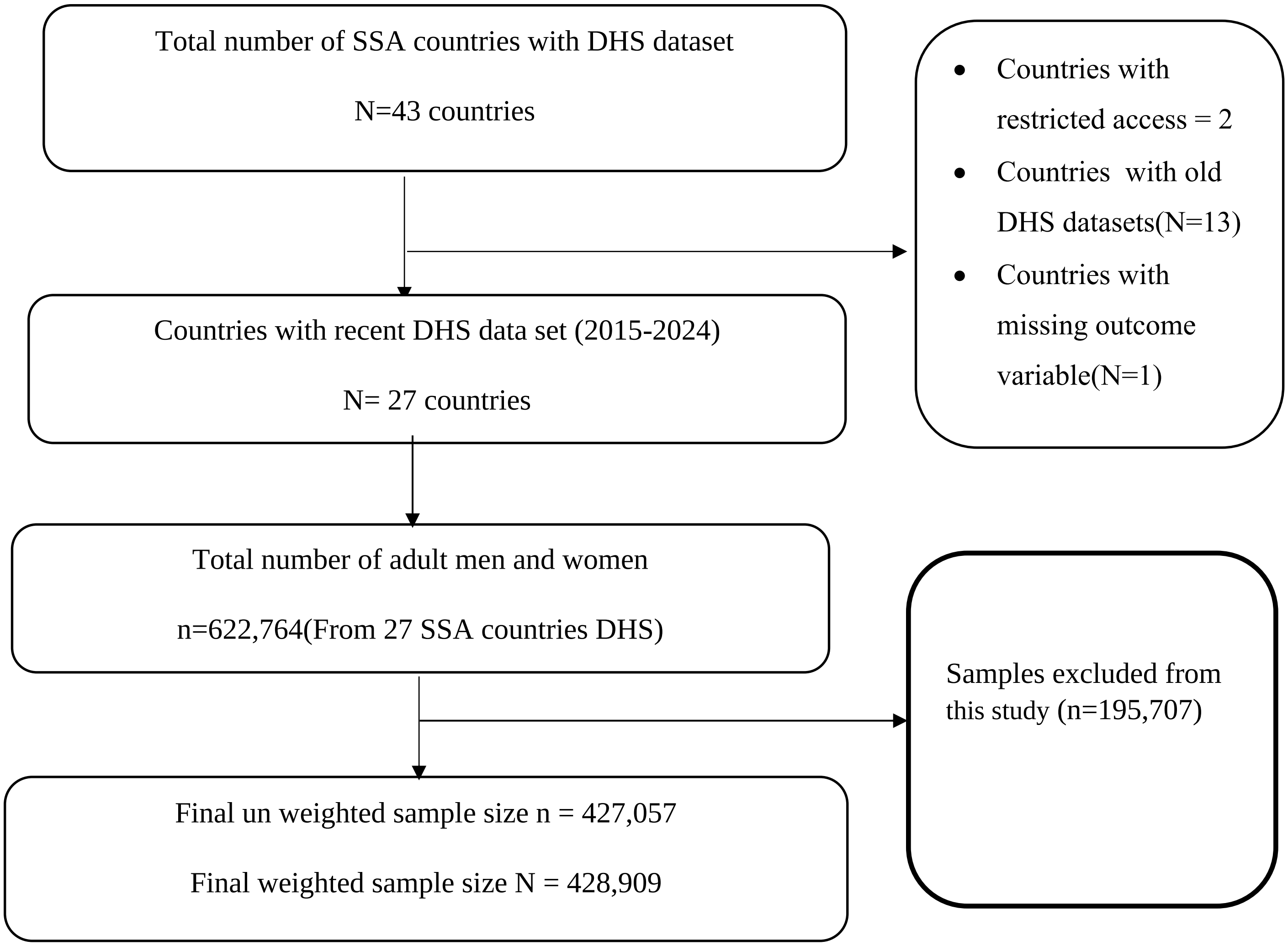

Figure: flow diagram of study sample selection from 27 SSA countries DHS data on pooled prevalence, spatial variation, associated factors and residence disparity of risky sexual behavior among sexually active men and women in SSA (2015-2024).

Supplementary information 4

Table: Socioeconomic, demographic, behavioral and clinical characteristics of participants from 2015 to 2024 (n= 428,909)

Variables	Category	Frequency (n)	Percent (%)	Risky sexual behavior	
				Yes (N/%)	No (N/%)
Education	No education	120,028	28%	9,396(8%)	110,632(92%)
	Primary	131,412	30.6%	19,769(15%)	111,643(85%)
	Secondary	142,732	33.3%	32,983(23%)	109,749(77%)
	Higher	34,737	8.1%	6,480(18.7%)	28,257(81.3%)
Age group	15-19	42,670	10%	16,735(39.2%)	25,935(60.8%)
	20-24	77,928	18.2%	18,651(23.9%)	59,277(76.1%)
	25-29	82,508	19.2%	12,395(15%)	70,113(85%)
	30-34	73,577	17.2%	7,821(10.6%)	65,756(89.4%)
	35-39	65,121	15.2%	5,770(8.9%)	59,351(91.1%)
	40-44	49,406	11.5%	4,181(8.5%)	45,225(91.5%)
	45-49	37,688	8.8%	3,074(8.2%)	39,624(91.8%)
Age at first sex	<18 years old	246,897	57.6%	45,329(18.4%)	201,568(81.6%)
	>= 18 years old	182,012	42.4%	23,299(12.8%)	158,713(87.2%)
Currently Working	Yes	302,711	70.6%	45,840(15.1%)	256,871(84.9%)
	No	127,198	29.4%	22,788(18.1%)	103,410(81.9%)
Wealth index	Poor	155,954	36.4%	21,905(14.1%)	134,049(85.9%)
	Middle	83,977	19.6%	13,721 (16.3%)	70,255(83.7%)
	Rich	188978	44%	33,002(17.5%)	155,977(82.5%)
Sex	Male	126,991	29.6%	25,340(20%)	101,651(80%)
	Female	301,918	70.4%	43,288(14.3%)	258,630(85.7%)
Marital status	Un married	103,259	24.1%	57,688(55.9%)	45,571(44.1%)
	Married	325,650	75.9%	10,939(3.4%)	314,711(96.6%)
Media exposure	Yes	240,633	56.1%	39742(16.5%)	200891(83.5%)
	No	188276	43.9%	28885(15.3%)	159391(84.7%)

Ever been tested for HIV	Yes	280,164	65.3%	37,782(13.5%)	242,382(86.5%)
	No	148,745	34.7%	30,846(20.7%)	147899(79.3%)

Supplementary information 5

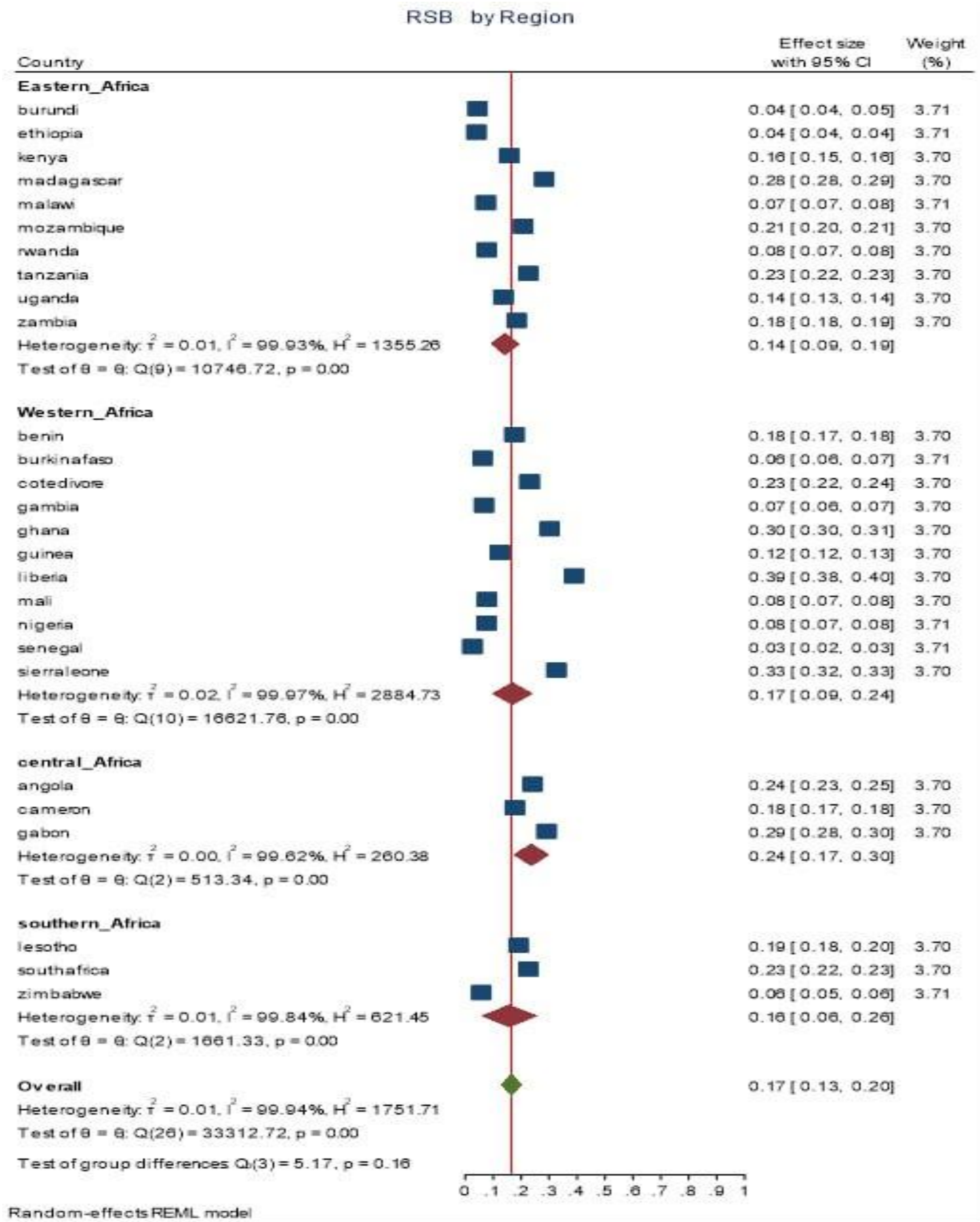

Figure: Forest plot of overall prevalence of risky sexual behavior in Sub Saharan Africa countries by region, from 2015-2024

Supplementary information 6

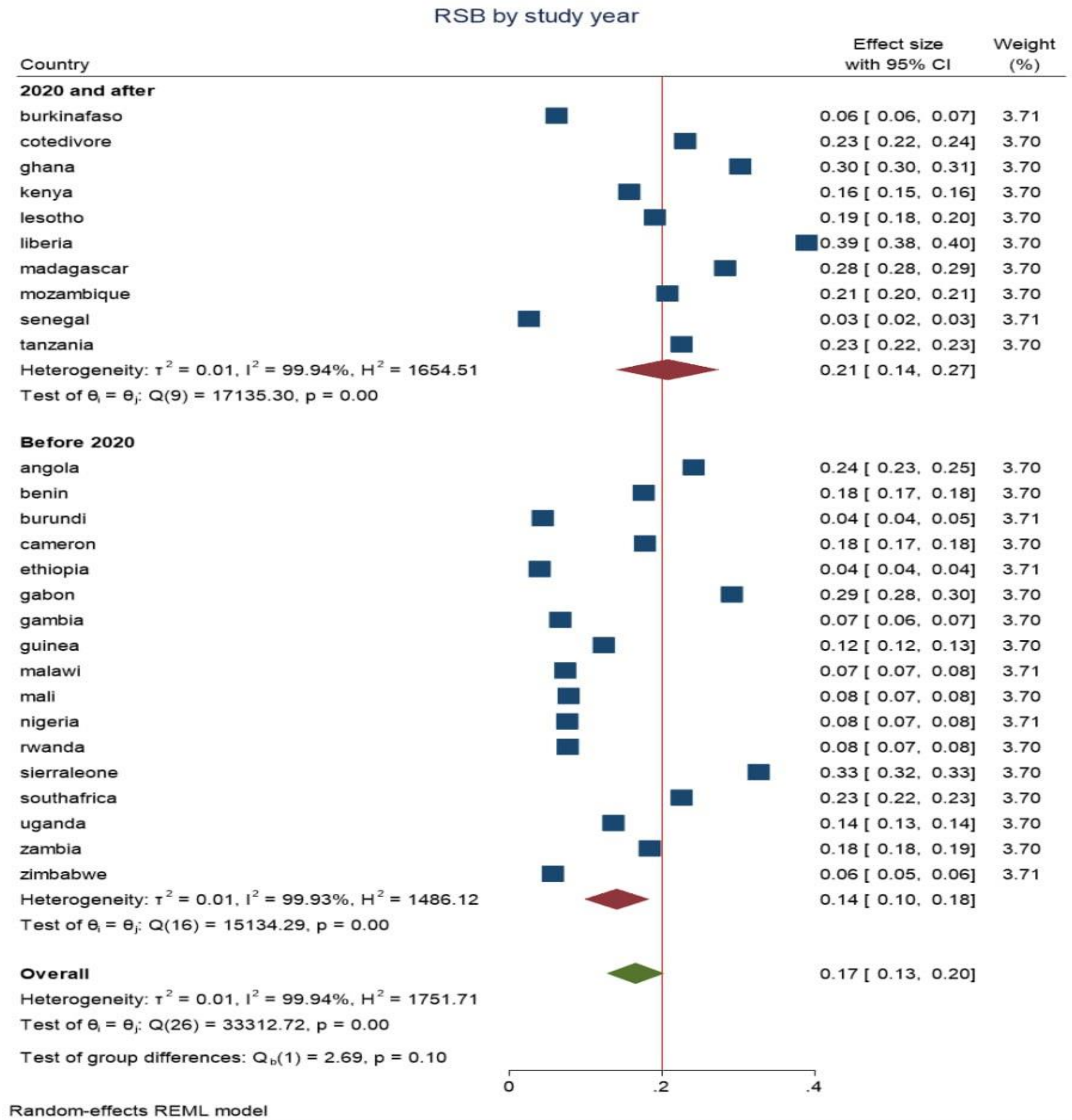

Figure: Forest plot of overall prevalence of risky sexual behavior in Sub Saharan Africa countries by year from 2015-2024

Supplementary information 7

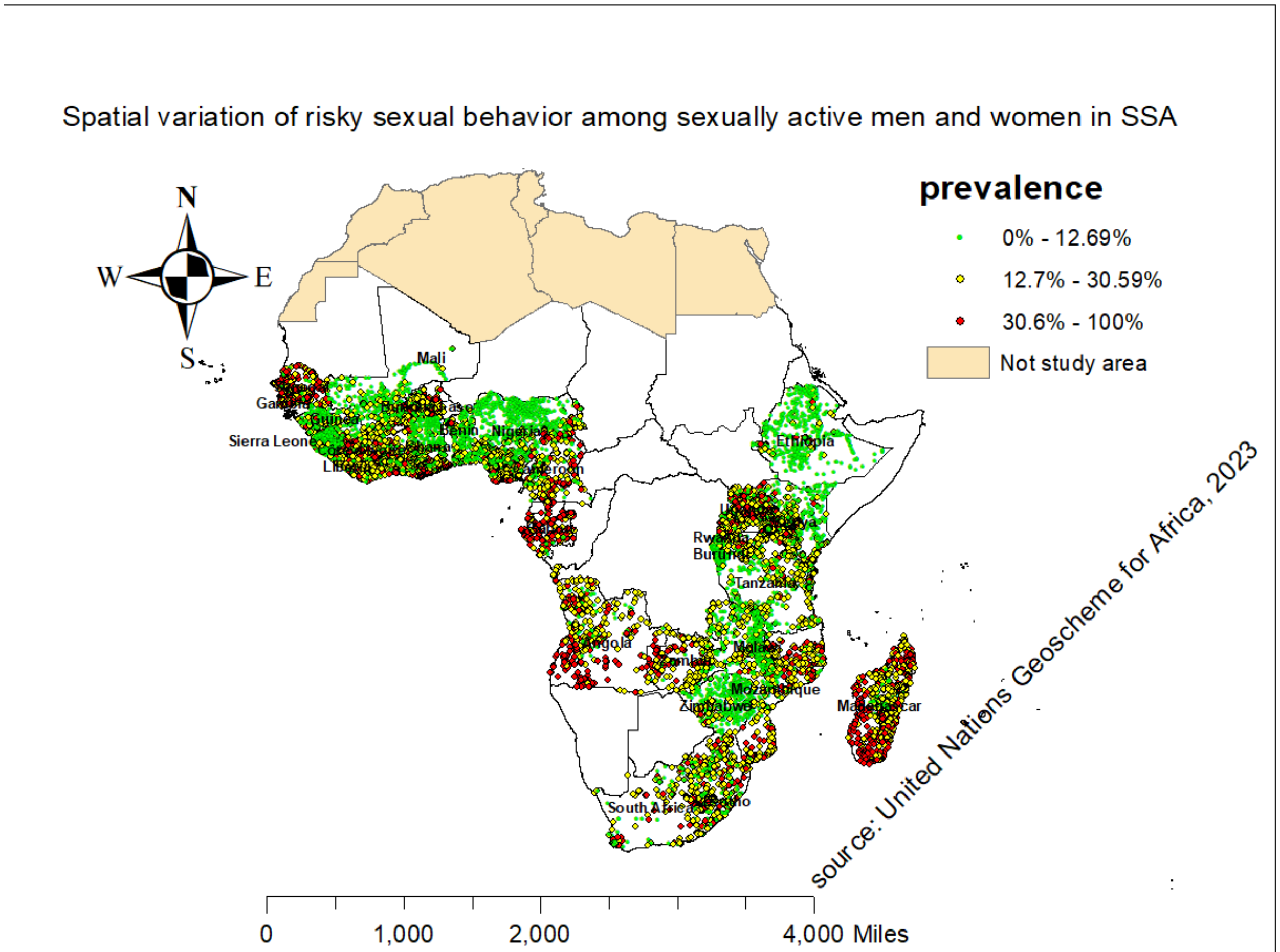

Figure: spatial variation of risky sexual behavior among sexually active men and women in sub-Saharan Africa, using recent DHS (2015-2024)

Supplementary information 8

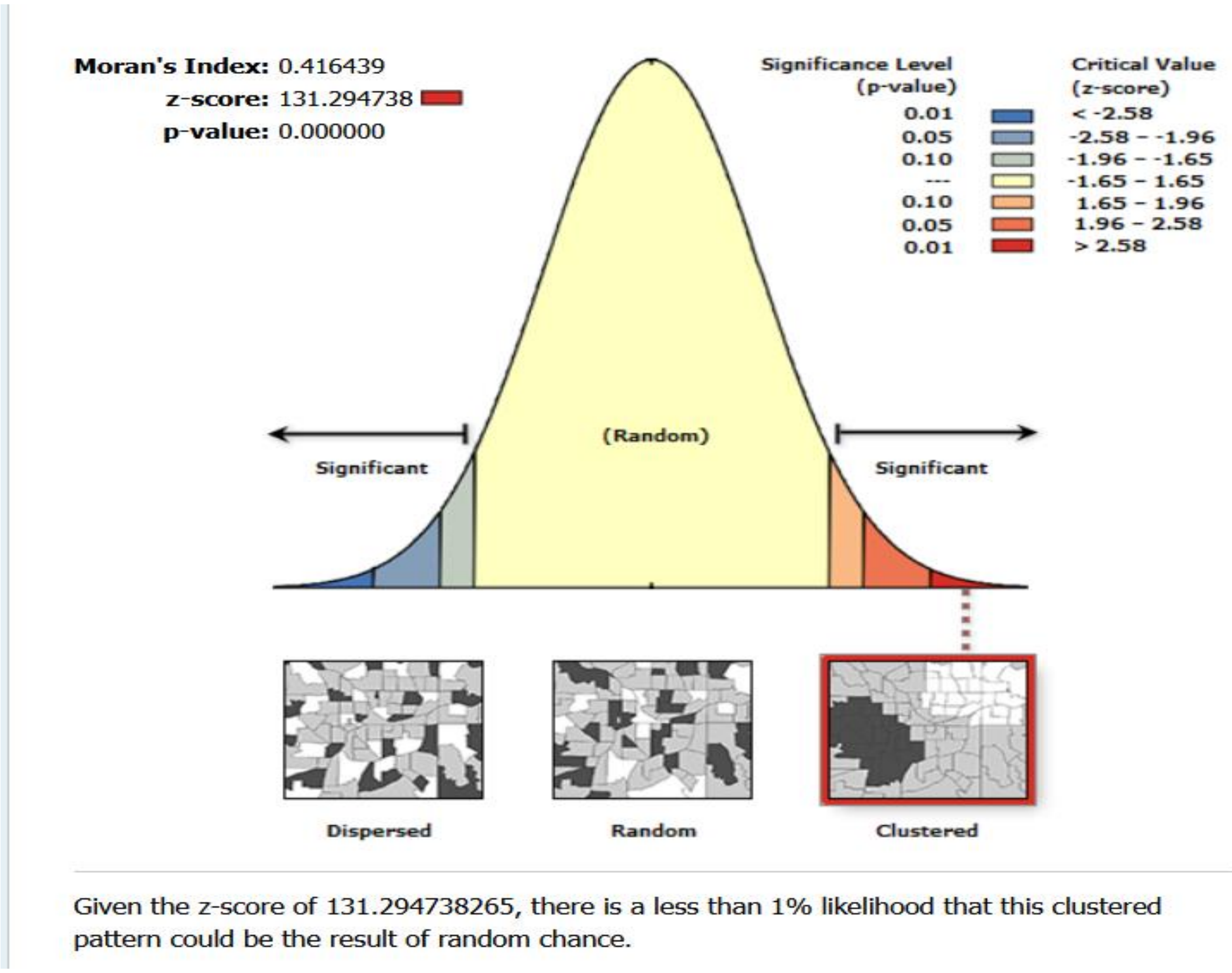

Figure: Spatial patterns of risky sexual behavior in sub-Saharan Africa, 2015-2024

Supplementary information 9

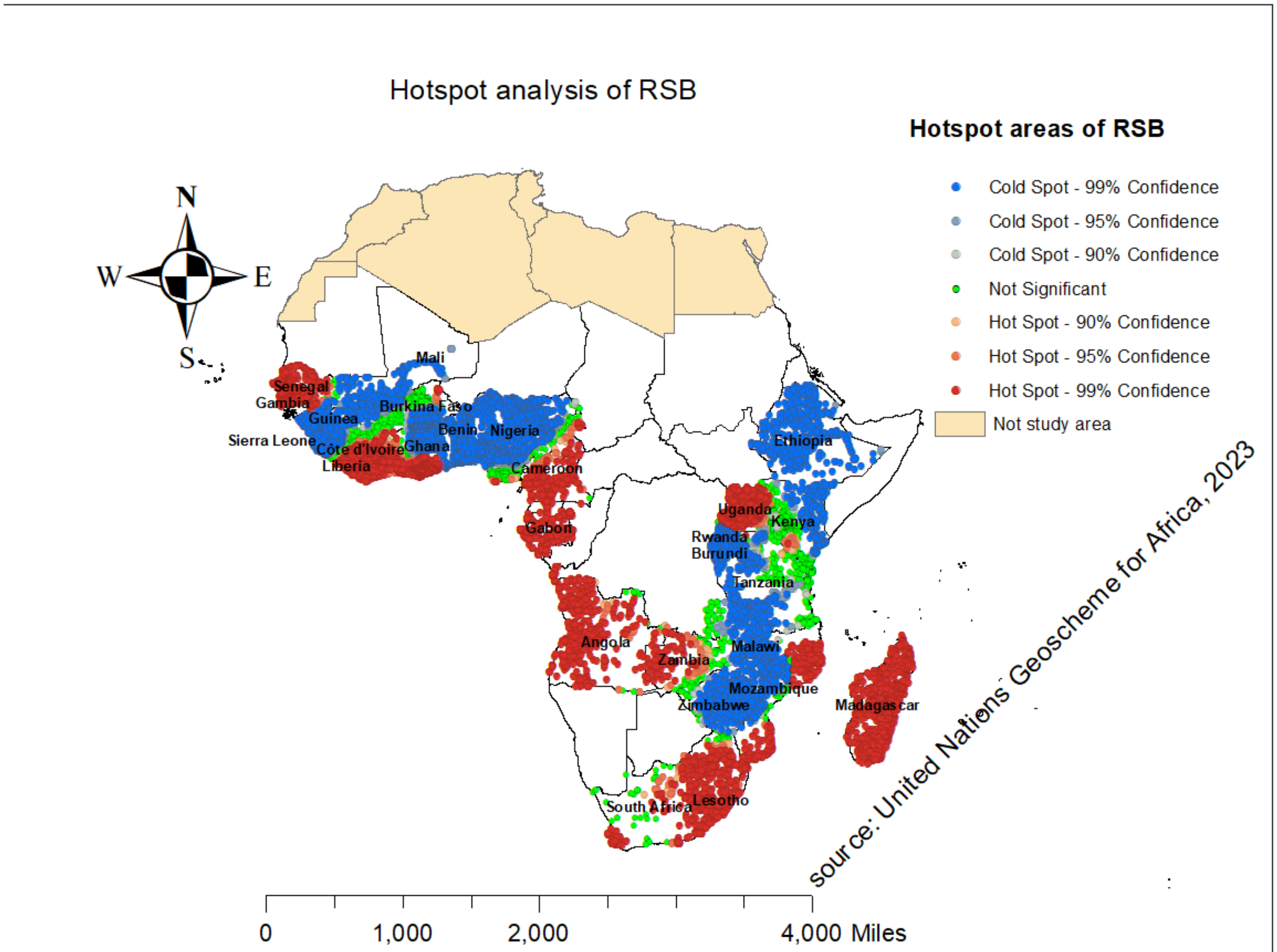

Figure: Hotspot analysis (Getis-Ord Gi*) statistic of risky sexual behavior among sexually active men and women

Supplementary information 10

Table: Sat Scan cluster analysis of risky sexual behavior among sexually active men and women in Sub Saharan Africa, 2015- 2024

Detected clusters	Number of included clusters	Coordinates/ radius	RR	LLR	P-value
1	656	(15.988095 N, 16.492659 W) / 506.78 km	2.21	2057.026823	<0.01
2	1523	(6.948997 S, 15.233172 E) / 1561.35 km	1.71	1425.880820	<0.01
3	379	(29.462799 S, 27.686337 E) / 120.25 km	2.34	1356.550076	<0.01
4	934	(24.179729 S, 47.227909 E) / 1451.29 km	1.79	1280.573082	<0.01
5	648	(2.999773 N, 30.896677 E) / 449.97 km	1.44	134.030921	<0.01
6	81	(10.340473 N, 15.231033 E) / 238.09 km	1.63	98.197232	<0.01
7	59	(5.337437 N, 0.625982 W) / 62.92 km	2.09	93.353226	<0.01

8	1158	(6.274853 N, 5.989029 W) / 532.23 km	1.15	57.161747	<0.01
9	26	(1.258803 S, 36.848667 E) / 6.83 km	1.69	50.263996	<0.01
10	41	(13.050715 N, 0.353991 W) / 88.88 km	1.57	27.825121	<0.01
11	51	(6.318276 N, 0.544612 E) / 90.92 km	1.61	27.073344	<0.01
12	27	(12.575741 S, 36.254082 E) / 171.24 km	1.69	21.415328	<0.01
13	2	(8.712307 N, 39.296909 E) / 19.08 km	2.42	19.559073	<0.01
14	3	(5.181980 S, 39.807789 E) / 3.65 km	3.49	17.796638	<0.01
15	1	(0.190380 N, 37.967262 E) / 0 km	4.17	17.382091	<0.01

Supplementary information 11

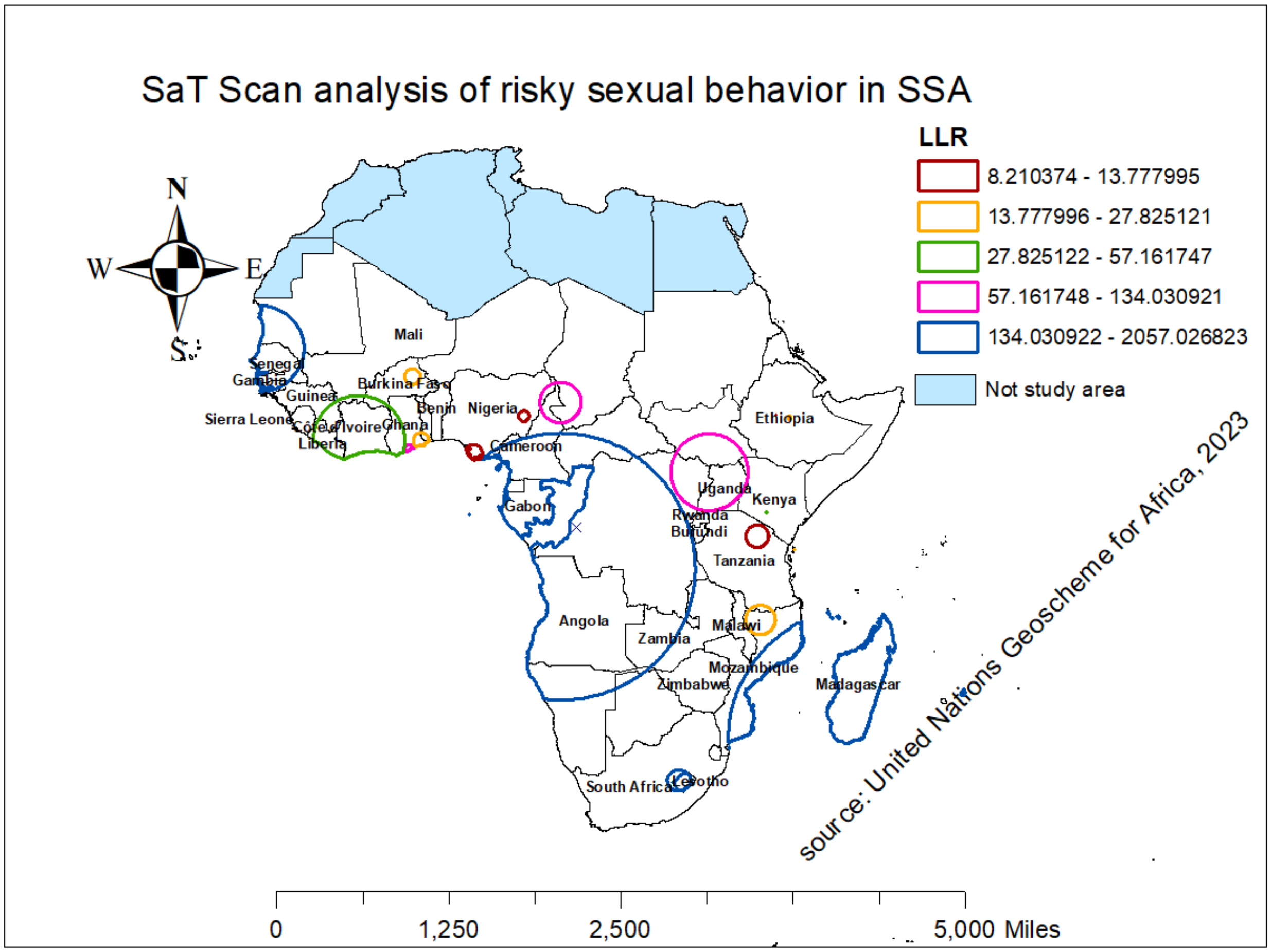

Figure: SaT Scan cluster analysis of risky sexual behavior among sexually active men and women in Sub Saharan Africa, 2015- 2024

Supplementary information 12

Table: Summary and model diagnostic outputs of OLS model fitting results for Risky sexual behavior among sexually active men and women in sub-Saharan Africa, 2015-2024

Variable	Coefficient (a)	StdError	t-Statistic	Probability (b)	Robust_SE	Robust_t	Robust_pr(b)	VIF(c)
Intercept	16.351886	0.128491	127.261216	<0.001	0.210829	77.559977	<0.001	------
Not Working	-0.156891	0.013247	-11.843179	<0.001	0.017453	-8.989405	<0.001	2.058767
HIV tested	-0.199430	0.008288	-24.062119	<0.001	0.012785	-15.598620	<0.001	2.767404
Male	-0.222828	0.020100	-11.085996	<0.001	0.029902	-7.451822	<0.001	3.482127
Not Married	1.398298	0.016090	86.902960	<0.001	0.043225	32.349462	<0.001	2.512367
Yes Media	-0.190472	0.009796	-19.443416	<0.001	0.015042	-12.662996	<0.001	3.748523

OLS diagnostics

Input features		Dependent variable	Risky sexual behavior
Number of observations	16176	Akaike's Information Criterion (AICc) [d]	124639.097710
Multiple R squared(d)	0.337180	Adjusted R-Squared [d]	0.336975

Joint F statistic(e)	1645.153604	Prob(>F), (5.16170) Degrees of freedom	<0.001
Joint Wald statistic(e)	1650.083146	Prob(>chi-squared), (5) degrees of freedom	<0.001
Koenker BP statistic(f)	1509.396847	Prob(>chi-squared), (5) degrees of freedom;	<0.001
Jarque -bera statistic(g)	12954.576237	Prob(>chi-squared), (2) degrees of freedom	<0.001
